# Modeling Wording Effects Does Not Help in Recovering Uncontaminated Person Scores: A Systematic Evaluation With Random Intercept Item Factor Analysis

**DOI:** 10.3389/fpsyg.2021.685326

**Published:** 2021-06-02

**Authors:** María Dolores Nieto, Luis Eduardo Garrido, Agustín Martínez-Molina, Francisco José Abad

**Affiliations:** ^1^Department of Psychology, Faculty of Life and Nature Sciences, Universidad Antonio deNebrija, Madrid, Spain; ^2^Department of Psychology, Pontificia Universidad Católica Madre y Maestra, Santiago de los Caballeros, Dominican Republic; ^3^Department of Social Psychology and Methodology, Faculty of Psychology, Universidad Autónoma de Madrid, Madrid, Spain

**Keywords:** method factor, wording effects, acquiescence, carelessness, factor scores, structural equation modeling

## Abstract

The item wording (or keying) effect consists of logically inconsistent answers to positively and negatively worded items that tap into similar (but polarly opposite) content. Previous research has shown that this effect can be successfully modeled through the random intercept item factor analysis (RIIFA) model, as evidenced by the improvements in the model fit in comparison to models that only contain substantive factors. However, little is known regarding the capability of this model in recovering the uncontaminated person scores. To address this issue, the study analyzes the performance of the RIIFA approach across three types of wording effects proposed in the literature: carelessness, item verification difficulty, and acquiescence. In the context of unidimensional substantive models, four independent variables were manipulated, using Monte Carlo methods: type of wording effect, amount of wording effect, sample size, and test length. The results corroborated previous findings by showing that the RIIFA models were consistently able to account for the variance in the data, attaining an excellent fit regardless of the amount of bias. Conversely, the models without the RIIFA factor produced increasingly a poorer fit with greater amounts of wording effects. Surprisingly, however, the RIIFA models were not able to better estimate the uncontaminated person scores for any type of wording effect in comparison to the substantive unidimensional models. The simulation results were then corroborated with an empirical dataset, examining the relationship between learning strategies and personality with grade point average in undergraduate studies. The apparently paradoxical findings regarding the model fit and the recovery of the person scores are explained, considering the properties of the factor models examined.

## Modeling Wording Effects Does Not Help in Recovering Uncontaminated Person Scores: a Systematic Evaluation With Random Intercept Item Factor Analysis

Most self-report scales in psychology often include both positively worded (PW) items, which are intended to measure the presence of a construct with a positive valence (e.g., extraversion), and negatively worded (NW) items, which measure the presence of a construct with a negative valence [e.g., introversion (Kam and Meyer, 2015a; Kam, 2016, 2018)]. The goal of this practice is usually to measure the two poles of the same construct. For example, a scale measuring extraversion may include several PW items (e.g., I make friends easily) as well as some NW items measuring introversion (e.g., I prefer to be alone), which tap the polar opposite ends of the construct. However, when both types of items are combined, respondents may manifest differential response styles to PW and NW items. This phenomenon is known as the item wording effect and consists of logically inconsistent answers to PW and NW items that tap into similar (but polar opposite) content (Kam and Meyer, 2015a; Kam, 2016).

For decades, the presence of different wording effects has been ubiquitous in psychological measurement (Jackson and Messick, 1958; Carmines and Zeller, 1979; Paulhus, 1991). An extensive body of research has demonstrated that wording effects may impact the psychometric properties of scales, deteriorating the model fit (Woods, 2006; Danner et al., 2015; Abad et al., 2018), spuriously increasing the dimensionality due to the emergence of separate factors for PW and NW items (Schmitt and Stults, 1985; Marsh, 1996; Barnette, 2000; Rodebaugh et al., 2004), reducing the reliability of measures (Schriesheim et al., 1991; Roszkowski and Soven, 2010), inflating or suppressing the structural relationships (Kam et al., 2012; Kam and Meyer, 2015b), and distorting the factor loading structures (Savalei and Falk, 2014; Navarro-González et al., 2016; Zhang et al., 2016).

However, the influence of wording effects on person score estimates has received much less attention. A possible reason is that most studies investigating wording effects are conducted, using real data collected in applied settings, making it impossible to know the uncontaminated true score of the respondents. In addition, prior simulation studies evaluating the recovery of person scores in the presence of response biases (e.g., Falk and Cai, 2016; Plieninger, 2016; Wetzel et al., 2016b) have been mainly focused on the influence of response styles, such as extreme responding (i.e., a tendency to select extreme response options). In general, few studies have systematically evaluated wording effects, and the ones that are available are frequently limited because they include only a single type of wording effect (Schmitt and Stults, 1985; Woods, 2006). Thus, the current literature lacks a systematic evaluation of the impacts that different wording effects may have, as well as of the conditions under which they are most harmful.

The random intercept item factor analysis (RIIFA) model (Billiet and McClendon, 2000; Maydeu-Olivares and Coffman, 2006) has shown to be a promising approach for modeling method variance due to wording effects over competing approaches (Savalei and Falk, 2014). First, it is very easy to implement in practice. Second, it generally produces substantial improvements in the model fit at the cost of only one degree of freedom in comparison to the “do-nothing” approach (i.e., fitting a model with only substantive factors, ignoring the presence of wording effects (Maydeu-Olivares and Coffman, 2006; Kam et al., 2012; Abad et al., 2018; Yang et al., 2018; Billiet and McClendon). Third, it is robust in recovering the substantive factor loading, even when its main assumption (i.e., equal method factor loading across all items) is violated (Savalei and Falk, 2014). Despite these positive characteristics, however, there is still limited knowledge about its performance in estimating certain parameters such as the uncontaminated person scores in the presence of wording effects.

In light of the aforementioned issues, the motivating goal of the study is to examine the impact of wording effects on parameter estimation, specifically person scores, in unidimensional data sets with categorical variables. To do so, we focused on three wording effects proposed in literature: *carelessness, item verification difficulty*, and *acquiescence* (Swain et al., 2008). Thus, the main aim of this study was to assess the performance of the RIIFA model in estimating person scores and other substantive parameters in the presence of wording effects, and to compare it with the “do-nothing approach.” The rest of this section is devoted to provide: (a) a conceptualization of the types of the wording effects considered in this study and the cognitive processes underlying them, (b) some examples of response patterns of the targeted wording effects, and (c) a description of the RIIFA model.

## Types of Wording Effects

A response bias is any systematic tendency to answer items irrespective of their content (Paulhus, 1991, 2002). Previous literature has usually distinguished between two types of response biases: response styles and response sets (Jackson and Messick, 1958). *Response styles* generally refer to a systematic tendency to use or avoid some specific response categories [e.g., extreme response style or the preference for extreme categories; e.g., Wetzel et al. (2016b)]. A number of studies have focused on demonstrating the stability of individual response styles across time and different constructs (e.g., Weijters et al., 2010a,b; Danner et al., 2015). In this regard, response styles have been traditionally conceptualized as response biases that are consistent across time and situations. In contrast, response sets have been defined as response biases that temporarily manifest in specific situations or settings [e.g., the tendency to provide a positive self-image in a personnel selection process (Jackson and Messick, 1958; Nunnally and Bernstein, 1994)]. Wetzel et al. (2016a), Van Vaerenbergh and Thomas (2012), and Ziegler (2015) provide further review of these response biases.

Within this conceptual framework, wording effects are another type of a response bias, which consists of logically inconsistent answers to PW and NW items that tap into similar (but polarly opposite) content (Kam and Meyer, 2015a; Kam, 2016). Building on the response process model developed in the survey research literature (Tourangeau et al., 2000), Swain et al. (2008) and Weijters and Baumgartner (2012) described the wording effects in terms of the cognitive processes underlying an item response. This model consists of four major steps: (a) comprehension (attending to the item and interpreting it), (b) retrieval (retrieving a relevant belief previously formed from long-term memory or transferring to working memory the information used to construct a new belief), (c) judgment (integrating the information retrieved previously and comparing it with the item representation), and (d) response (representing the answer to the given scale and producing a response).

### Carelessness

Several terms have been used to refer to a pattern of responding in which respondents pay insufficient attention to the items' content, such as random responding (Meade and Craig, 2012), noncontingent responding (Baumgartner and Steenkamp, 2001), inattentiveness (Johnson, 2005), or insufficient effort responding (Huang et al., 2012). The concept of carelessness has been broadly used to refer to different random or nonrandom response patterns such as fully or partially random responding, using the same response category (i.e., straight-line responding) or response sequence, or skipping items (e.g., Johnson, 2005; Swain et al., 2008; Meade and Craig, 2012).

A pervasive type of carelessness is the systematic (non-random) type in which a respondent may answer according to the expectations that he or she has formed about what is being measured according to the questionnaire instructions or the content of the initial items (Schmitt and Stults, 1985; Woods, 2006; Weijters et al., 2013). This type of carelessness occurs at the initial step of the response process model (Tourangeau et al., 2000) during the comprehension phase (Swain et al., 2008; Weijters and Baumgartner, 2012; Weijters et al., 2013). Authors suggesting this variant of carelessness usually associate it with misresponses to the NW items. This is often built on the assumption that respondents may generate the expectation that items are stated affirmatively based on everyday experiences with language and on the results from prior studies, showing that most Likert type items are affirmations (Swain et al., 2008). However, we argue that these reasons do not necessarily imply that misresponses due to carelessness will only occur to the NW items. For example, if the questionnaire instructions explicitly reveal that a construct with negative valence is being measured (e.g., burnout), a careless respondent may assume that items will be NW and he or she might fail in responding to the PW items.

Previous research investigating carelessness has mainly focused on the detection of careless respondents through the use of different methods, such as instructed response items, indices based on repeated responses (e.g., long-string), and factor mixture modeling (e.g., Meade and Craig, 2012; Kam and Meyer, 2015a; Kam and Fan, 2018). Some simulation studies have examined the impact of systematic carelessness (to NW items) on the factor structure of unidimensional scales. For example, Schmitt and Stults (1985) found out that only 10% of careless respondents were necessary for the emergence of a spurious second dimension (these authors used principal component analysis). Also, Woods (2006) reached similar conclusions in the context of confirmatory factor analysis: With only 10% of carelessness respondents, a two-factor model presented a better model fit and thus was preferred to the unidimensional solution.

### Item Verification Difficulty

Swain et al. (2008) conceptualized item verification difficulty as a type of inconsistent responding that occurs when a respondent's belief in the construct being measured (i.e., his or her true trait level) mismatches the item content during the judgment phase of the response process model (Tourangeau et al., 2000). Swain et al. (2008) suggested that the item verification process can be explained according to the constituent-comparison model (Carpenter and Just, 1975). This model postulates that a respondent's difficulty to verify an item and thus the probability of misresponding it will depend on the complexity of comparing his/her own belief or true trait level on the construct being measured to the item content. This difficulty will depend on whether the item content is on the same pole (i.e., is truth) or on the opposite pole (i.e., is false) relative to the respondent's belief (i.e., true trait level), and whether it is affirmed or negated. According to the constituent-comparison model, for example, a person who believes that he or she is introverted (i.e., has a low trait level in extraversion) will have *increasing difficulty* in responding to the following items: “I am introverted” (true affirmation), “I am extroverted” (false affirmation), “I am not introverted” (false negation), and “I am not extroverted” (true negation). This model implies that a respondent will have to perform more cognitive operations to compare an item with his or her belief as the difficulty of such comparison increases.

Previous studies have suggested that wording effects may be related to reading ability. The studies of Marsh (1986, 1996) showed how method effects (in this case, associated with NW items) were weaker for more verbally able students. In addition, Swain et al. (2008) confirmed through a series of experiments the item verification predictions made by the constituent-comparison model: inconsistent responding and difficulty to process statements linearly increased with true affirmations, false affirmations, false negations, and true negations.

### Acquiescence

Acquiescence is the tendency to respond to items using agree categories (i.e., the positive side of the scale) irrespective of their content (e.g., Paulhus, 1991; Weijters et al., 2013; Wetzel et al., 2016a). This wording effect influences the response phase (Swain et al., 2008; Weijters and Baumgartner, 2012; Weijters et al., 2013), which is the final step of the response process model (Tourangeau et al., 2000). Knowles and Condon (1999) suggest that the cognitive process underlying acquiescence can be explained according to the dual-process model of understanding (Gilbert, 1991). This model posits that, initially, a respondent automatically accepts the item content (comprehension phase), and, subsequently, the respondent can reevaluate it in order to decide whether to reject it or continue accepting it (reconsideration phase). This second step implies an effort for the participant, so it can be omitted, depending on the ability and motivation of the respondent. If this occurs, the respondent will automatically agree to all items, irrespective if they are PW or NW, manifesting an acquiescent response pattern (Swain et al., 2008; Weijters and Baumgartner, 2012).

Previous studies examining the effects of acquiescence mostly did it from an empirical perspective through the computation of different measures based on the endorsement of polar opposite items (e.g., Hinz et al., 2007; Rammstedt and Farmer, 2013) or many items with heterogeneous content (Baumgartner and Steenkamp, 2001; Weijters et al., 2013). However, some of these measures (i.e., those that are not based on heterogeneous items) may also reflect other wording effects such as carelessness (Weijters et al., 2013; Kam and Meyer, 2015a), leading to erroneous conclusions about the influence of acquiescence. In contrast, very few studies have examined the impact of acquiescence from the perspective of Monte Carlo simulation. Grønhaug and Heide (1992) simulated acquiescent responses to Likert type items and found out that inconsistent responses might distort results from regression and factor analysis. More recently, Plieninger (2016) has found out that the impact of acquiescence on reliability, validity, and scale scores estimates was greater in unbalanced scales with fewer NW items.

### Illustration of Wording Effects Response Patterns

Table 1 presents some examples of response patterns that examinees with low (top section) or high trait levels (bottom section) may show when responding to a scale with 10 items (five PWs marked as “+,” and five NWs marked as “−”). In each case, both non-reversed (left section) and reversed responses (right section) are presented. The first row always represents the uncontaminated true pattern. All response patterns correspond to a hypothetical examinee that misresponds to 50% of the items according to different wording effects: carelessness to NW items (i.e., responses to NW items were reversed), item verification difficulty (i.e., responses to PW/NW items were reversed for a person with a true low-/high-trait level), and acquiescence (i.e., responses to PW/NW items were replaced by categories implying agreement for a person with a true low-/high-trait level). Looking at the total raw scores (computed with reversed item responses), it can be seen that, in general, total scores for respondents with low (high) true trait levels will be upwardly (downwardly biased) in the presence of any wording effect.

**Table 1:** Examples of response patterns for the wording effects of carelessness, item verification difficulty, and acquiescence.

	**Non-reversed responses**										**Reversed-responses**										
**Type of wording effect**	**+**	**+**	**+**	**+**	**+**	**-**	**-**	**-**	**-**	**-**	**+**	**+**	**+**	**+**	**+**	**-**	**-**	**-**	**-**	**-**	**Sum**
**Low trait level**																					
None	1	1	1	2	2	3	3	4	4	4	1	1	1	2	2	2	2	1	1	1	14
Carelessness to NW items	1	1	1	2	2	2	2	1	1	1	1	1	1	2	2	3	3	4	4	4	**25**
Item verification difficulty	4	4	4	3	3	3	3	4	4	4	4	4	4	3	3	2	2	1	1	1	**25**
Acquiescence	3	3	3	4	4	3	3	4	4	4	3	3	3	4	4	2	2	1	1	1	**24**
**High trait level**																					
None	4	4	4	3	3	2	2	1	1	1	4	4	4	3	3	3	3	4	4	4	36
Carelessness to NW items	4	4	4	3	3	3	3	4	4	4	4	4	4	3	3	2	2	1	1	1	**25**
Item verification difficulty	4	4	4	3	3	3	3	4	4	4	4	4	4	3	3	2	2	1	1	1	**25**
Acquiescence	4	4	4	3	3	4	4	3	3	3	4	4	4	3	3	1	1	2	2	2	**26**

*The values in the table correspond to items with a 5-point Likert response scale. Inconsistent answers appear highlighted in gray. Overestimated person scores are shown in boldface, whereas underestimated person scores are shown in underlined italics. A positive (+) denotes a positively worded item and a negative sign (–) a negatively worded item*.

It should be noted that different wording effects might produce indistinguishable observable response patterns in practice (Weijters et al., 2013; Kam and Meyer, 2015a). For example, looking at the non-reversed responses in Table 1, two persons with a high true trait level may present a similar response pattern if one of them responds as if all items were PW (carelessness to NW items), and the other one has problems to process NW items (item verification difficulty). Wording effects might also be confounded under other circumstances not illustrated in Table 1. For example, some types of acquiescent respondents that systematically use the highest agree category might resemble some types of careless respondents (e.g., one displaying a straight-line responding pattern), and vice versa. However, there is an important difference in the response process: Careless respondents overlook item content (the problem arises at the initial comprehension phase), whereas acquiescent ones pay attention to it (the problem occurs at the final response phase (Weijters et al., 2013; Kam and Meyer, 2015a).

## The RIIFA Model

Maydeu-Olivares and Coffman (2006) introduced the RIIFA model as an extension of the common factor model that allows for the explicit modeling of consistent individual differences in the use of the response scale. In the common factor model, the response of participant *j* to item *i* (*y*_*ij*_) can be written as:

(1) $$\begin{array}{l} y_{ij}=\mu_{i}+\lambda_{i}\prime f_{j}+e_{ij}, \end{array}$$

where μ_*i*_ is the intercept for item *i*, λ_*i*_ is the vector of factor loading for item *i*, **f**_*j*_ is the vector of substantive factor scores for participant *j*, and *e*_*ij*_ is the error term for participant *j* on item *i*. Assuming that the mean of the common factors and the error terms is zero, and that the error terms are uncorrelated with each other and with the common factors, the covariance matrix implied by this model (Σ_*y*_) is expressed as

(2) $$\begin{array}{l} \sum_{y}=\Lambda \Psi \Lambda \prime +\Theta , \end{array}$$

where *lambda* (Λ) is a *m* × *r* matrix of factor loading for *m* variables and *r* common factors, *psi* (Ψ) is a *r* × *r* covariance matrix of the common factors, and *theta* (Θ) is a *m* × *m* covariance matrix of the error terms.

In the RIIFA model, the intercept (γ_*ij*_) is decomposed into a fixed part (μ_*i*_), common to all respondents but differing across items, and a random part (ζ_*j*_), common to all items but differing across respondents:

(3) $$\begin{array}{l} y_{ij}=\gamma_{ij}+\lambda_{i}\prime f_{j}+e_{ij},\gamma_{ij}=\mu_{i}+\zeta_{j} \end{array}$$

(4) $$\begin{array}{l} \;y_{ij}=\mu_{i}+\zeta_{j}+\lambda_{i}\prime f_{j}+e_{ij} \end{array}$$

If in addition to the previous assumptions of the common factor model it is assumed that the term ζ_*j*_ is standardized and that it is uncorrelated with the error terms and with the common factors, the covariance structure implied by the RIIFA model can be written as

(5) $$\begin{array}{l} \sum_{y}=1\omega 1\prime +\Lambda \Psi \Lambda \prime +\Theta , \end{array}$$

where ω is the variance of ζ_*j*_ across all the respondents.

In the RIIFA model, the parameter to be estimated is ω and not the random intercept for each examinee. To do so, it is only necessary to define an additional method factor in the common factor model in which all the unstandardized factor loadings are fixed to 1 (if items are not reverse coded), and ω is left free to be estimated.

Prior research has shown that wording effects can be successfully modeled through the RIIFA model, as evidenced by the improvements in the model fit, in comparison to models that only contain substantive factors (e.g., Billiet and McClendon, 2000; Maydeu-Olivares and Coffman, 2006; Kam et al., 2012; Abad et al., 2018; Yang et al., 2018; Schmalbach et al., 2020). Besides, the RIIFA model has been shown to enhance the discriminant validity of scales (Kam et al., 2012). Also, Savalei and Falk (2014) and de la Fuente and Abad (2020) evaluated its behavior to estimate item parameters when respondents make an idiosyncratic use of response scale with unidimensional structures. They found out that the RIIFA model was superior to competing approaches (including the “do-nothing” approach) and robust to the violation of its assumption of equal wording factor loading across items.

## Purpose of the Current Study

A principal concern regarding the use of self-report measures is the potential influence of wording effects on responses of the examinees. Therefore, the main motivating goal of this study was to examine the impact of different types of wording effects—carelessness, item verification difficulty, and acquiescence—on person score estimates. In addition, we also evaluated the impact of the different types of the wording effects on other parameters of interest, such as the model fit, factor loading, and structural validity, for models composed of one substantive factor. Model estimates resulting from the traditional one-factor model (the “do-nothing” approach) were compared to those obtained from the RIIFA model. Finally, we analyzed an empirical dataset examining the relationship between learning strategies and personality with grade point average in undergraduate studies to ascertain the impact of wording effects, and their handling, on the different model properties.

This study has three main unique features. First, the comprehensive evaluation of the recovery of person scores and its relationship with other parameter estimates in the presence of different wording effects. Second, the inclusion for the first time of the item verification difficulty wording effect to be examined *via* Monte Carlo methods. Third, the systematic evaluation of the RIIFA model to estimate person scores (and other parameters not previously studied with this model) in the presence of different wording effects.

## Study 1: Impact of Carelessness on Parameter Estimation

In this study, Monte Carlo methods were employed to systematically assess the impact of the wording effect of carelessness on the performance of the 1F and RIIFA models.

## Method

### Study Design

Three independent variables were systematically manipulated: the amount of wording effect, the sample size, and the test length. These variables have been shown to affect the performance of factor analysis methods with categorical variables (Woods, 2006; Forero et al., 2009; Garrido et al., 2011, 2013).

1. Amount of wording effect (PERC.WE): This variable indicates the percentage of items (out of the total number of items in the test) that each inconsistent examinee misresponded to. Five levels were manipulated: 10, 20, 30, 40, and 50%. The condition of absence of wording effect (PERC.WE = 0%) was included as a baseline with which to compare.
2. Sample size (N). Three levels were included-−200, 500, and 1,000—to represent a small, a medium, and a large number of cases, respectively, for the factor analysis of categorical variables (Muthén and Kaplan, 1985; Forero et al., 2009; Savalei and Rhemtulla, 2013).
3. Test length (T.LENG). Three levels were included with 12, 24, and 60 items to measure the substantive construct, which may represent a short fixed-length test, a long fixed-length test, and a large item pool, respectively.

In total, the 6 × 3 × 3 (PERC.WE × N × T.LENG) factorial design produced 54 factor combinations; for each of which, 100 sample replicates were generated.

### Data Generation and Models Evaluated

**Figure 1:**
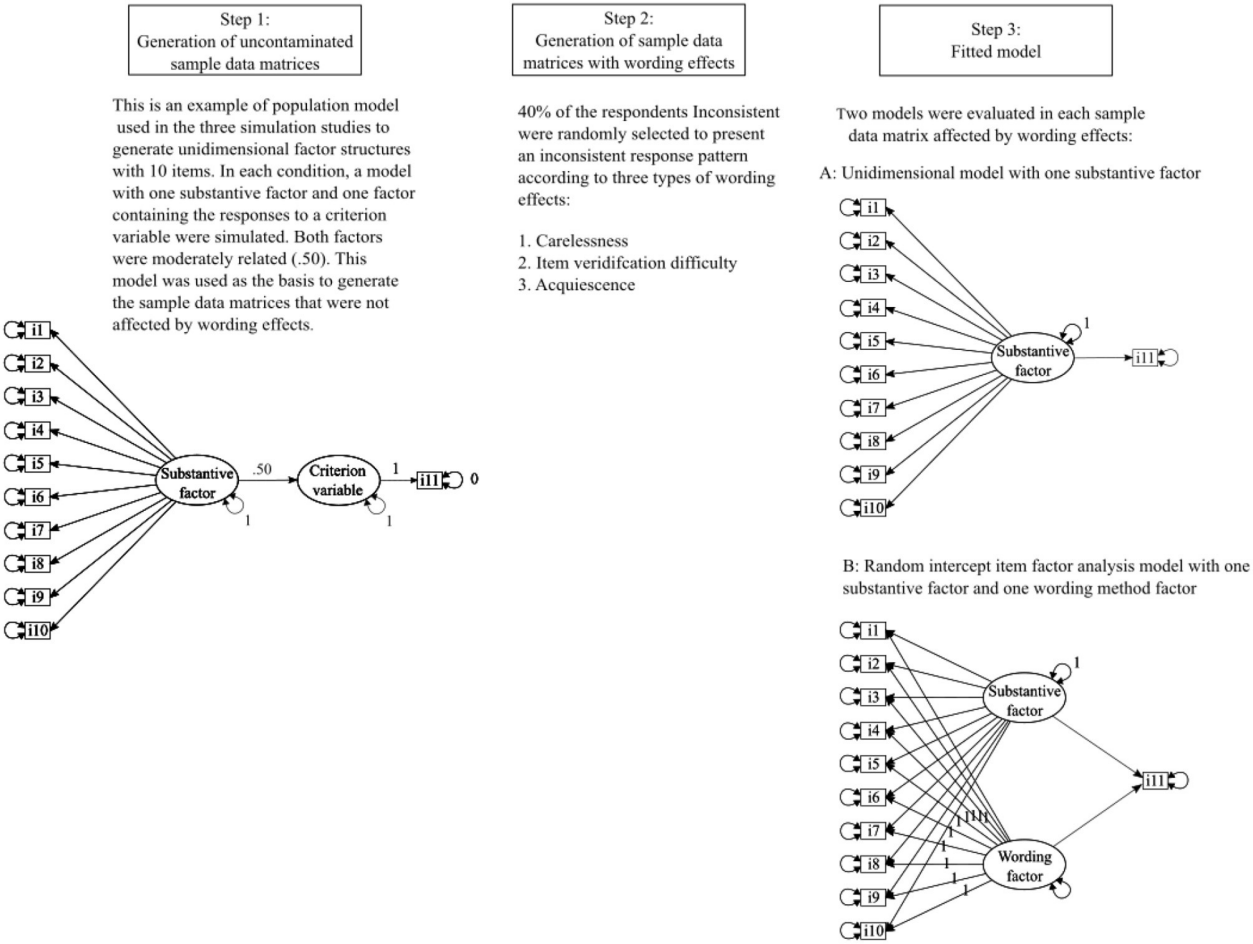
Flowchart diagram describing the main steps of the three simulation studies.

Figure 1 presents a flowchart illustrating the main steps of the simulation study. The simulation study involved three steps: (1) generation of the uncontaminated sample data matrices, (2) generation of the sample data matrices with wording effects, and (3) estimation of the fitted models.

#### Step 1: Generation of Uncontaminated Sample Data Matrices

For each of the nine simulated conditions without wording effects, 100 uncontaminated (i.e., without WE) sample data matrices of symmetrically distributed categorical variables with four response options were generated. Data matrices were generated according to the bidimensional model showed in Step 1 of Figure 1, which contained one substantive factor representing the responses to the construct of interest and another factor representing the responses to a criterion variable (i.e., a criterion variable factor, henceforth). Regarding the substantive factor, half of the items were conceptualized as PW and the other half as NW items (i.e., balanced scales). The example illustrates a 10-item (five PWs, five NWs) test. In all the conditions, the mean substantive factor loading was fixed to 0.70, and loadings were randomly drawn from a uniform distribution with values ranging from 0.60 to 0.80 to generate items with variable factor loadings. Then, half of the simulated factor loadings were randomly assigned a negative sign to simulate the factor loadings of the NW items. Additionally, the criterion variable factor was simulated by generating the responses to an item with a standardized loading of 1.0 on such a factor. The substantive factor and the criterion variable correlated strongly (*r* = 0.50) according to Cohen (1988).

Sample data matrices were simulated according to the common factor model procedure described next. First, the reproduced population correlation matrix (with communalities in the diagonal) was computed:

(6) $$\begin{array}{l} R_{R}=\Lambda \Phi \Lambda \prime , \end{array}$$

where **R**_**R**_ is the reproduced population correlation matrix, *lambda* (Λ) is the measurement model (i.e., a *k* × 2 factor loading matrix for *k* variables and two factors: the substantive factor and the criterion variable factor), and *phi* (Φ) is the structure matrix of the latent variables (i.e., a 2 × 2 matrix of correlations among the substantive factor and the criterion variable factor).

The population correlation matrix **R**_**P**_ was then obtained by inserting unities in the diagonal of **R**_**R**_, thereby raising the matrix to full rank. The next step was performing Cholesky decomposition of **R**_**P**_, such that

(7) $$\begin{array}{l} {\text{R}}_{\text{P}}={\text{U}}^{\prime }\text{U} \end{array}$$

Subsequently, the sample data matrix of continuous variables was computed as

(8) $$\begin{array}{l} \text{X}=\text{Z}\text{U}, \end{array}$$

where **Z** is a matrix of random standard normal deviates with rows equal to the sample size and columns equal to the number of variables.

The resulting continuous variables were categorized (except the criterion variable, which was not included in the following steps) by applying the following threshold values so that they had symmetrical distributions: −1.5, 0, and 1.5 (Garrido et al., 2011, 2013).

#### Step 2: Generation of Sample Data Matrices With Wording Effects

Wording effects were generated by introducing inconsistent responses in the uncontaminated sample data matrices previously simulated (step 2 in Figure 1). To do so, 40% of the simulees were randomly selected to present inconsistent responses. Then, following previous research (Schmitt and Stults, 1985; Woods, 2006), carelessness response patterns were simulated by reversing the answers to NW items (1 = 4, 2 = 3, 3 = 2, and 4 = 1) for each inconsistent respondent according to the desired amount of wording effects in each case. For each uncontaminated sample data matrix, five sample data matrices with wording effects were generated according to the five levels of PERC.WE established. Table 1 includes some examples of response patterns for hypothetical examinees responding carelessly to NW items. These “respondents” were postulated to respond inconsistently to 50% of the items of a 10-item test. We decided to simulate carelessness to NW items arbitrarily, based on previous studies. This would follow a real-life scenario where the first items of a test were PW and/or the examinee had an idea that the trait being measured had a positive valence. Nevertheless, simulating carelessness to the PW items would have yielded the same general conclusions.

#### Step 3: Estimation of the Fitted Models

The two structural equation models represented in step 3 of Figure 1 were estimated for each of the simulated sample data matrices. The first model (Figure 1, step 3, A) had one substantive factor measured by the simulated target (categorical) items, and the (continuous) item representing the observed scores for the simulees on a criterion variable that was regressed on the substantive factor. As the main core of this model is the traditional one-factor model with a substantive factor, we will refer to this model as 1F. The second model (Figure 1, step 3, B) included the RIIFA approach to model one substantive factor and one method factor to control for wording effects (Billiet and McClendon, 2000; Maydeu-Olivares and Coffman, 2006), and the observed criterion variable that was regressed on the substantive factor and the wording factor. In the RIIFA model, the loadings in the wording factor were fixed to 1 because sample data matrices contained unrecoded item scores, and the variance of the wording method factor was estimated. As the main core of this second model is the estimation of the RIIFA, we will refer to it as “RIIFA.” Both models were estimated, using robust-weighted least squares estimation based on a matrix of polychoric correlations [WLSVM, see Muthén and Muthén (2012)].

### Assessment Criteria

Although the primary estimates of interest were the substantive factor scores, the performance of each model was also evaluated according to three other fundamental aspects in model validation: model fit, recovery of substantive factor loadings, and structural validity. For each model, the following assessment criteria were obtained.

#### Model Fit

It was evaluated according to the root mean square error of approximation (RMSEA) and the comparative fit index (CFI). For the CFI, values of 0.90 or greater indicate an acceptable fit and values of 0.95 or greater represent a good fit, whereas RMSEA values between 0.05 and 0.08 are indicative of an acceptable model fit, and values below 0.05 represent a good fit (Hu and Bentler, 1999; McDonald and Ho, 2002).

#### Recovery of the Substantive Factor Loadings

It was evaluated for PW and NW items separately by computing the mean bias error (MBE) and the mean absolute error (MAE) in each case:

(9) $$\begin{array}{l} MBE=\;\frac{\sum \left(\hat{\lambda }-\lambda \right)}{k}, \end{array}$$

(10) $$\begin{array}{l} MAE=\frac{\sum |\left(\hat{\lambda }-\lambda \right)|}{k}, \end{array}$$

where *k* is the number of positive or negative items, $\hat{\lambda }$ is the estimated loading on the substantive factor, and λ is the true loading on the substantive factor.

An MBE of 0 reflects a total lack of bias, whereas negative and positive MBE values indicate that loadings were underestimated and overestimated in absolute value, respectively, for positive items and the opposite for negative items. For the MAE, higher values signal larger biases in estimating the true factor loadings, while a value of 0 indicates that the factor loadings are estimated with perfect accuracy.

#### Recovery of the Substantive Scores

It was evaluated with the correlation between the uncontaminated substantive factor scores (estimated by applying the 1F model to the sample data matrix without wording effects) and the contaminated factor scores that were estimated by applying each model to the data matrix with wording effects. A mixed analysis of variance (ANOVA) was performed in order to evaluate the differences between the 1F and RIIFA models in the recovery of the uncontaminated substantive scores across the manipulated conditions. The dependent variable was the total recovery of the substantive factor scores, the repeated measures within-subjects independent variable was the models (1F, RIIFA), and the between-subjects independent variables were the amount of wording effects, the sample size, and the test length. Due to the low convergence rate of the RIIFA model with 10% of wording effects across the three studies, the cases for that condition were not included in these analyses. Only those higher order interactions with large or near-large-effects sizes were interpreted. According to Cohen (1988), partial eta squared (${\eta_{p}}^{2}$) values of 0.01, 0.06, and 0.14 or greater represent small, medium, and large effects, respectively.

#### Structural Validity

It was evaluated through the magnitude of the regression coefficients associated with the substantive factor and the wording factor (only for the RIIFA model) that explained the continuous (uncontaminated criterion) variable, as well as the proportion of variance explained by the model (*R*^2^).

The programs used to run the simulation and estimate the factor models were developed with *Mplus* 7 (Muthén and Muthén, 2012) and the R package MplusAutomation (Hallquist and Wiley, 2018). The ANOVA statistical analyses and the simulation were performed using SPSS (Version 23) and R (R Core Team, 2018), respectively.

## Results

### Convergence Rates

The convergence rates reported in this section indicate for each model tested (1F, RIIFA) the proportion of estimated solutions that produced simultaneously the fit statistics, the matrix of factor loadings, the factor scores, the regression coefficients, and the *R*^2^. The convergence rate of the 1F model was always 100%. The overall convergence rate for the RIIFA model was 92.71%. Nonconvergence occurred with low amounts of wording effects (10 or 20%) and the tests with 10 or 20 items. In those conditions, convergence rates improved with larger tests and higher amounts of wording effects: 26.67% (PERC.WE = 10%, 10 items), 71.00% (PERC.WE = 10%, 20 items), and 93.00% (PERC.WE = 20%, 10 items).

### Model Fit

**Figure 2:**
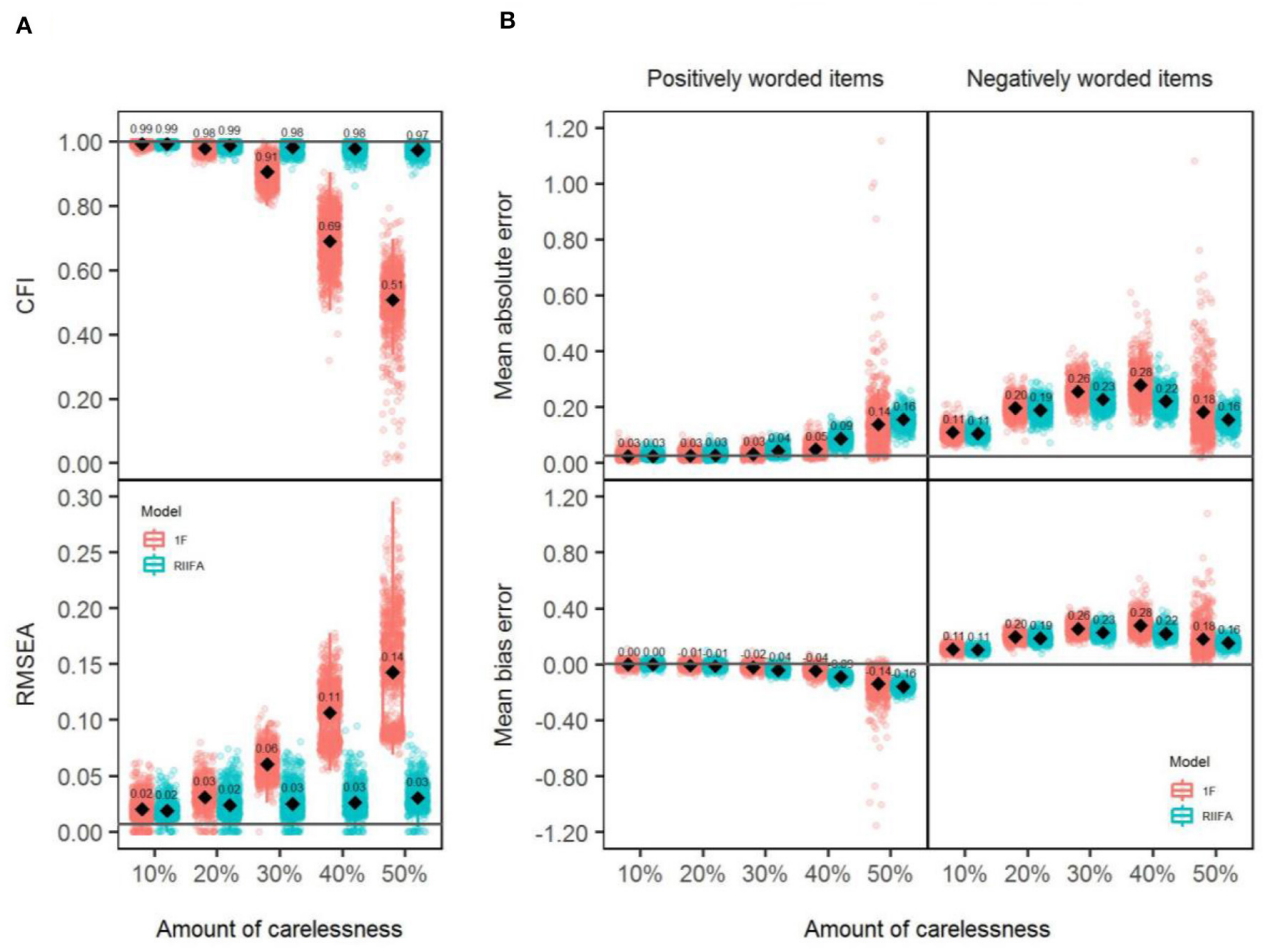
Model fit and recovery of substantive factor loadings for the unidimensional (1F) model and the random intercept item factor analysis (RIIFA) model in the presence of carelessness. 1F, unidimensional model with one substantive factor; RIIFA, random intercept item factor analysis model with one substantive factor and one wording method factor. In **(A)**, the horizontal gray lines represent the mean CFI and RMSEA values for the condition with 0% of wording effect. In **(B)**, the horizontal gray lines represent the mean absolute error and the mean bias error for the condition with 0% of wording effect.

Panel A of Figure 2 shows the CFI and RMSEA values obtained with both models through all the simulated sample data matrices across different amounts of wording effects. With lower amounts of wording effects (particularly 10%), both models showed an excellent model fit, presenting always the CFI and RMSEA mean values ($\bar{CFI},\;\bar{RMSEA}$), very close to 1 and 0, respectively. However, as the amount of wording effects increased, the differences between models were more notable: The 1F model gradually presented a poorer fit, and the values of the fit indices progressively departed from an acceptable fit, reaching the worst values with PERC.WE = 50% ($\bar{CFI}$ = 0.51, $\bar{RMSEA}$ = 0.14). In contrast, the RIIFA model showed an almost perfect model fit with any amount of wording effects.

### Recovery of the Substantive Factor Loadings

Panel B of Figure 2 shows the individual MBEs and MAEs for the simulated sample data matrices obtained with both models (1F, RIIFA) for each type of item (PW, NW) across the different amounts of wording effects. Looking at the average MAE ($\bar{MAE}$) values, in general, both models produced less accurate estimations for NW than for PW items across conditions, except with 50% of misresponded items where both models performed similarly and also produced similar estimates between them (e.g., for NW items, $\bar{MAE}$[1F] = 0.18 and $\bar{MAE}$[RIIFA] = 0.16) and for both types of items (e.g., for the RIIFA model, $\bar{MAE}$[PW] ≈ $\bar{MAE}$[NW] = 0.16). Moreover, estimates with both models were gradually less precise as the percentage of misresponded items increased. The only exception was found for NW items when the amount of wording effect grew from 40 to 50%: In this condition, the $\bar{MAE}$ decreased markedly from 0.28 to 0.18 for the 1F model and from 0.22 to 0.16 for the RIIFA model. Besides, a look at the MBE values revealed that both models tended to underestimate the factor loadings of any type of item, and that this tendency increased with higher amounts of wording effect.

### Recovery of the Substantive Factor Scores

**Figure 3:**
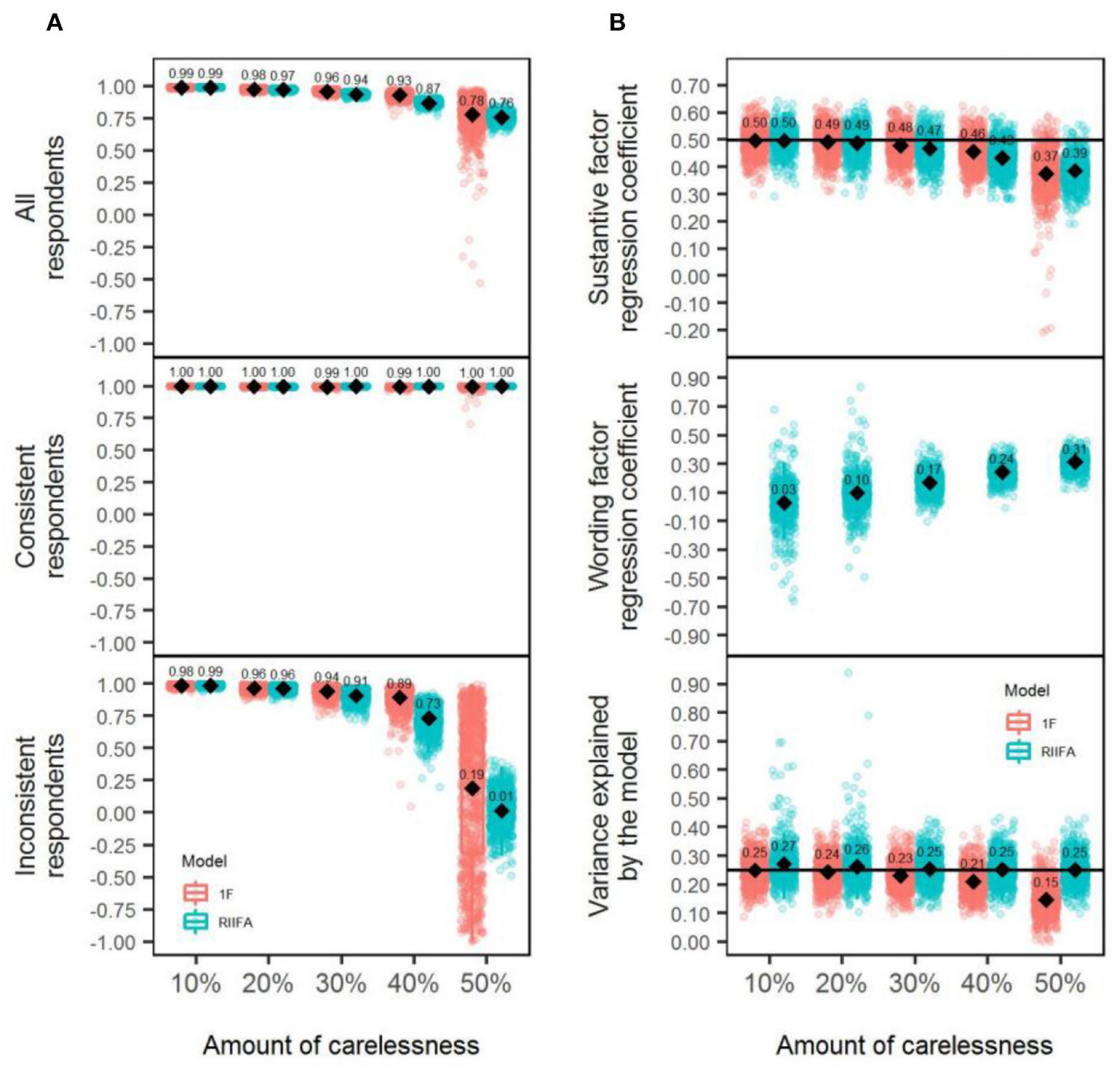
Recovery of substantive factor scores and estimation of structural validity with the 1F model and the RIIFA model in the presence of carelessness. In **(A)**, represented values are Pearson correlations, and results of the recovery of uncontaminated scores are presented for all the respondents, separately for consistent respondents, and separately for inconsistent respondents. 1F, unidimensional model with one substantive factor; RIIFA, random intercept item factor analysis model with one substantive factor and one wording method factor. In **(B)**, the horizontal black lines represent the substantive factor regression coefficient and the variance explained by the model with 0% of wording effect.

To better understand the performance of both models, the recovery was evaluated by computing the correlation between the uncontaminated and contaminated factor scores in three ways for each simulated sample data matrix: (a) considering the scores for all the respondents (consistent and inconsistent; henceforth, “Total recovery”), (b) considering consistent respondents scores exclusively (henceforth “Recovery for consistent respondents”), and (c) considering inconsistent respondents scores exclusively (henceforth, “Recovery for inconsistent respondents”). Results from the mixed ANOVA (Table 2, the carelessness column) revealed that a large effect size (${\eta_{p}}^{2}$ = 0.17, *p* < 0.001) was associated with the differences in performance between models in favor of the 1F model, which showed to be slightly superior to the RIIFA across conditions (overall, the mean correlation, $\bar{r}$, was 0.93 and 0.90, respectively, for the 1F and RIIFA model, respectively). Almost all interactions displayed ${\eta_{p}}^{2}$ values lower or equal to 0.02. Only the two-way interaction model × amount of wording effects showed a near-large-effect size (${\eta_{p}}^{2}$ = 0.11, *p* < 0.001). This interaction is depicted in the upper section of panel A in Figure 3 and shows that both models performed almost similarly with 20% of carelessness, whereas, with 50% of carelessness, the 1F model ($\bar{r}=\;$0.78) proved to be slightly superior to the RIIFA ($\bar{r}$ = 0.76) in recovering the uncontaminated person scores. The performance of both models gradually deteriorated as the amount of wording effect increased.

**Table 2:** Mixed analysis of variance effect sizes for the wording effects of carelessness, item verification difficulty, and acquiescence.

**Effect type/variables**	**Carelessness**	**Item verification difficulty**	**Acquiescence**
**Main effects**			
Model	0.17	0.14	0.20
**Two-way interactions**			
Model × Amount of wording effects	0.11	0.05	0.24
Model × Sample size	0	0	0.02
Model × Test length	0	0.02	0
**Three-way interactions**			
Model × Amount of wording effects × Sample size	0.01	0.01	0.04
Model × Amount of wording effects × Test length	0.02	0.02	0.01
Model × Sample size × Test length	0	0	0
**Four-way interaction**			
Model × Amount of wording effects × Sample size × Test length	0	0	0

*Tabled values are partial eta squared ($\eta_{p}^{2}$) estimates of variance explained by each of the effects shown. The dependent variable was the correlation between the uncontaminated substantive scores and the substantive scores from the contaminated datasets. Large effect sizes ($\eta_{p}^{2}$ = 0.14) are bolded and underlined*.

The middle and lower sections of panel A in Figure 3 show the recovery of substantive factor scores for the inconsistent and consistent respondents, respectively, across the two-way interaction model × amount of wording effects. Regarding the consistent respondents, both models always estimated with perfect accuracy the substantive scores of the consistent respondents (the mean correlation between the uncontaminated and contaminated scores was always 1.00). Looking at the results for the inconsistent respondents, the patterns showed by both models mirrored the results previously described for the total recovery, but the $\bar{r}$ values were systematically lower. It should be noted that the recovery for these respondents with 50% of wording effects was especially poor if looking at the average ($\bar{r}$[1F] = 0.19, $\bar{r}$[RIIFA] = 0.01). This might be explained, because, if a person misresponds to all the items in one direction (all PW or all NW), it is impossible to recover his/her uncontaminated score because there is no way to know if the correct score is what he/she responded to PW items or what he/she answered to the NW items. In other words, the answers of this person to both types of items are equally consistent.

**Figure 4:**
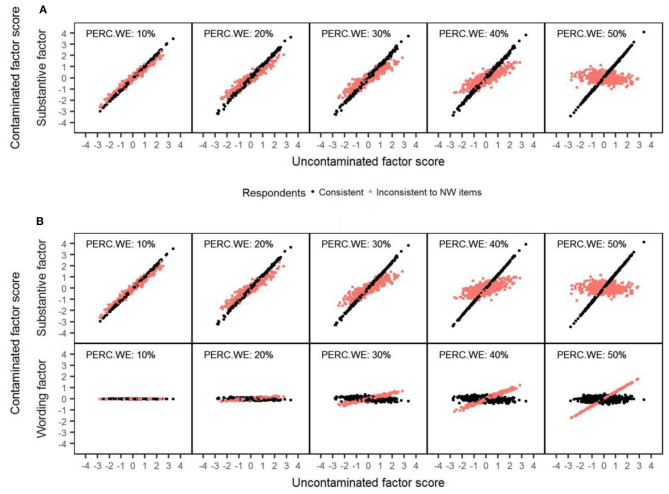
Example of recovery of the substantive factor scores with the 1F and RIIFA models in the presence of carelessness. The data represented corresponds to simulated unidimensional data sets with 1,000 respondents and 20 variables. 1F, unidimensional model with one substantive factor; RIIFA, random intercept item factor analysis model with one substantive factor and one wording method factor; PERC.WE, amount of wording effect; NW, negatively worded. **(A)** 1F model. **(B)** RIIFA model.

To better understand the previous results, Figure 4 shows for each model a series of scatterplots to illustrate the relationship between the uncontaminated and contaminated substantive scores as the amount of wording effects increased. To do so, we simulated a sample data matrix with 1,000 respondents and 20 items, which was later modified according to the levels of carelessness established. As shown before, for consistent respondents (colored in black) the substantive scores are always estimated with total precision with both models because they delineate a perfect diagonal straight line. In the case of inconsistent respondents (colored in red), with both models, the contaminated scores for respondents that had low uncontaminated scores tend to be increasingly biased upward, whereas the contaminated scores for respondents with high uncontaminated scores are progressively biased downward. This displacement is progressively more noticeable as the percentage of misresponded items is higher. Figure 4 also presents the correlation between the uncontaminated substantive scores and the estimated wording factor scores for the RIIFA model. Overall, consistent respondents had wording scores of medium magnitude (i.e., around 0) independently of the value of their uncontaminated substantive scores. Regarding inconsistent respondents, the estimated wording scores were increasingly correlated in a positive way with the substantive scores as the amount of wording effects was greater. This means that wording scores increasingly reflect the uncontaminated trait level of these examinees as more items are answered inconsistently.

### Structural Validity

The panel B of Figure 3 shows the regression coefficients associated with the substantive factor for both models and the wording factor of the RIIFA, as well as the proportion of explained variance by each model (*R*^2^). In terms of the mean regression coefficient for the substantive factor, both models showed a tendency to produce downwardly biased estimates, on average, with higher amounts of wording effects. In addition, the regression coefficient associated with the wording factor showed a tendency to increase gradually as the amount of carelessness grew, reaching non-negligible values in the conditions of greater carelessness. This might explain why the mean proportion of variance explained by the model was moderately greater for the RIIFA model in comparison to the 1F model. Indeed, it should be noted that the RIIFA model always reproduced the same amount of variance (on average) as the model fitted in the dataset without wording effects.

## Study 2: Impact of Item Verification Difficulty on Parameter Estimation

In this study, Monte Carlo methods were employed to systematically assess the impact of the wording effect of item verification difficulty in the performance of the 1F and RIIFA models.

## Method

The study design and the procedure followed to generate the uncontaminated sample data matrices (see Figure 1) were the same as the one described in Study 1. However, in this case, inconsistent respondents were simulated by reversing the answers (1 = 4, 2 = 3, 3 = 2, and 4 = 1) to PW items if the uncontaminated substantive score of a respondent was below 0, or to NW items if the uncontaminated substantive score of a respondent was above 0. That is, it was assumed that a person responded correctly to true affirmations and responded incorrectly to false affirmations. When the PERC.WE was below 50%, item responses were randomly selected and reversed until the desired amount of wording effects (% of items answered inconsistently) for each respondent was reached. The proportion of respondents in each database that answered inconsistently was again fixed at 40%. Finally, the same assessment criteria of Study 1 were obtained to evaluate the performance of both models.

## Results

### Convergence Rates

As in Study 1, only the RIIFA model showed nonconvergence solutions, with an overall convergence rate of 93.38%. The pattern of convergence rates was similar to that found in Study 1: nonconvergence occurred with low amounts of wording effects (10 or 20%) and the tests with 10 or 20 items. Convergence rates improved with larger tests and higher amounts of wording effects: 30.33% (PERC.WE = 10%, 10 items), 93.67% (PERC.WE = 10%, 20 items), and 77% (PERC.WE = 20%, 10 items).

### Model Fit

**Figure 5:**
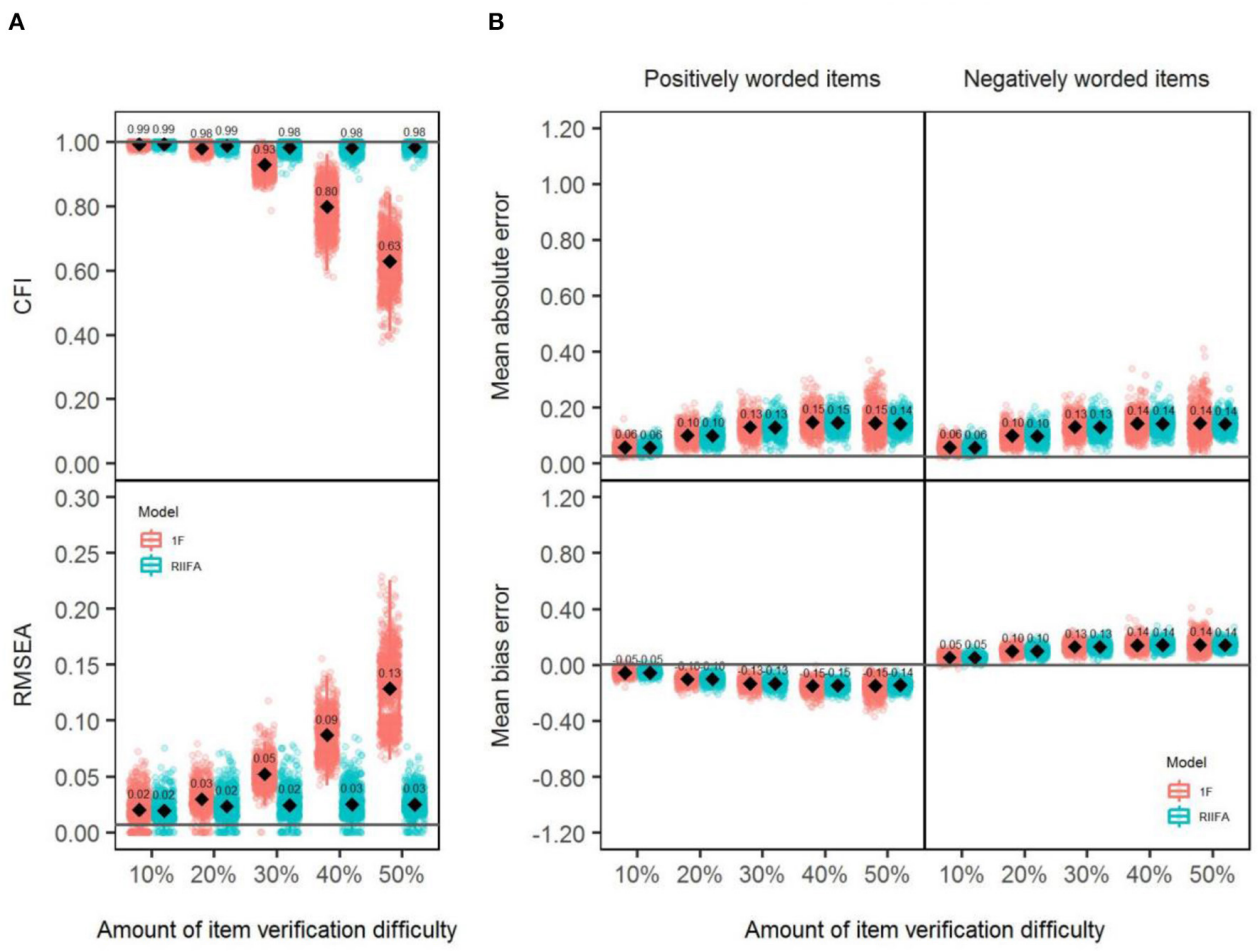
Model fit and recovery of substantive factor loadings for the 1F model and the RIIFA model in the presence of item verification difficulty. 1F, unidimensional model with one substantive factor; RIIFA, random intercept item factor analysis model with one substantive factor and one wording method factor. In **(A)**, the horizontal gray lines represent the mean CFI and RMSEA values for the condition with 0% of wording effect. In **(B)**, the horizontal gray lines represent the mean absolute error and the mean bias error for the condition with 0% of wording effect.

Panel A of Figure 5 shows the CFI and RMSEA values obtained with both models through all the simulated sample data matrices across different amounts of wording effects. Results revealed a similar trend similar to that found in Study 1: Both models performed similarly, showing a perfect fit, with lower amounts of item verification difficulty. However, as the amount of wording effect increased, the differences were more notable in favor of the RIIFA model, which consistently showed an excellent fit, whereas the 1F model gradually presented a poorer fit.

### Recovery of the Substantive Factor Loadings

Panel B of Figure 5 shows the individual MBEs and MAEs for the simulated sample data matrices obtained with both models (1F, RIIFA) for each type of item (PW, NW) across the different amounts of wording effects. Looking at the $\bar{MAE}$, in general, both models produced similar estimates between them and for both types of items for any amount of wording effects. As in Study 1, in general, the estimates with both models were gradually less precise as the percentage of misresponded items increased, except for the 50% PERC.WE condition, which was similar to the 40% condition. As in Study 1, the MBE values showed that both models tended to underestimate the factor loadings of any type of item, and that this tendency increased with higher amounts of wording effects.

### Recovery of the Substantive Factor Scores

**Figure 6:**
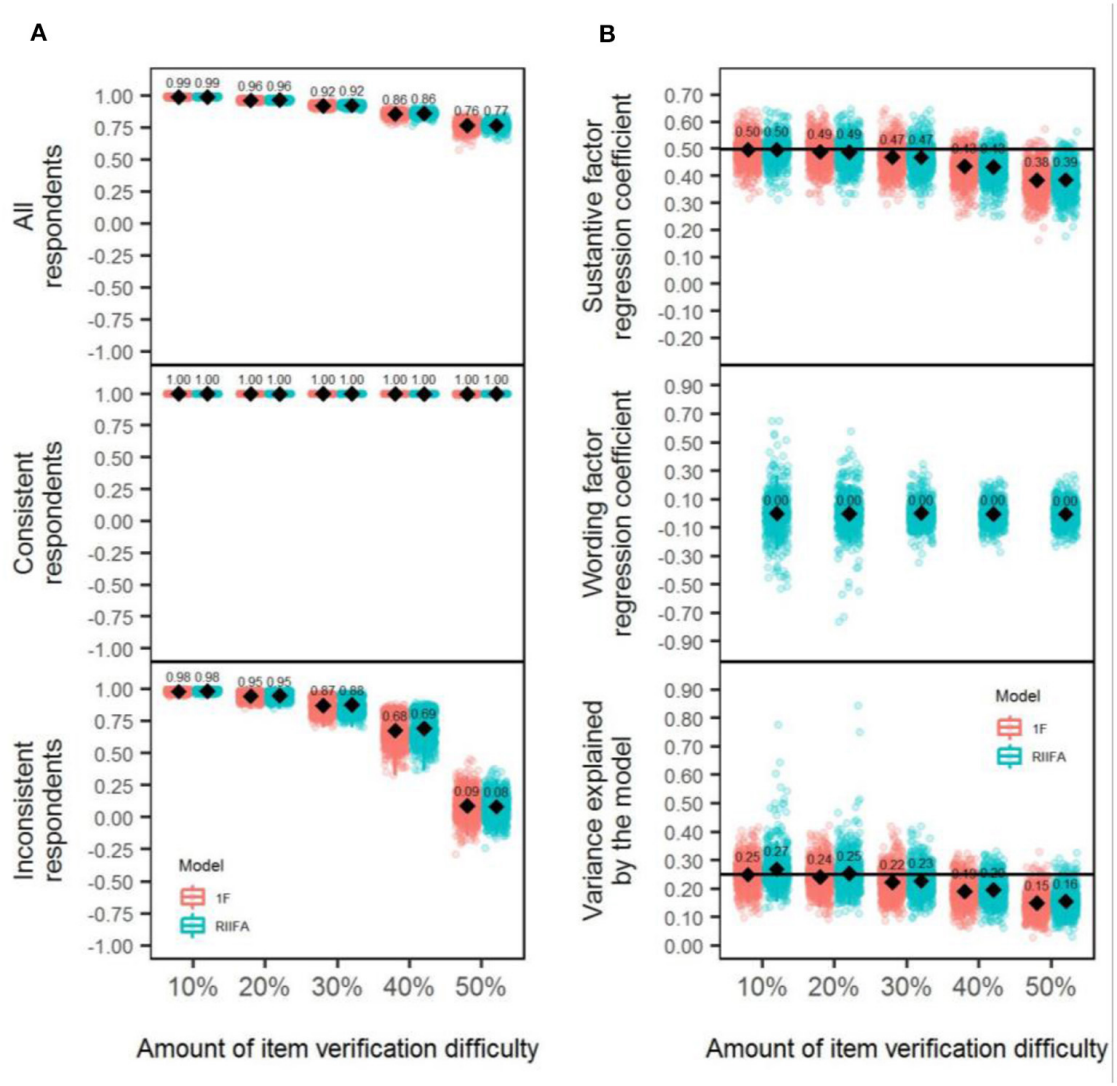
Recovery of substantive factor scores and estimation of structural validity with the 1F model and the RIIFA model in the presence of item verification difficulty. In **(A)**, represented values are Pearson correlations, and results of the recovery of uncontaminated scores are presented for all the respondents, separately for consistent respondents, and separately for inconsistent respondents. 1F, unidimensional model with one substantive factor; RIIFA, random intercept item factor analysis model with one substantive factor and one wording method factor. In **(B)**, the horizontal black lines represent the substantive factor regression coefficient and the variance explained by the model with 0% of wording effect.

The results of the mixed ANOVAs (Table 2, column item verification difficulty) comparing the precision of the factor score estimates across the manipulated conditions for the 1F and RIIFA models showed that, although a large effect size (${\eta_{p}}^{2}$ = 0.14, *p* < 0.001) was associated with the differences in performance between models, it had no practical relevance because, on average, the overall recovery was similar for both models (0.9 for the 1F and 0.89 for the RIIFA). This large effect size emerged because of the low variability of the individual results for the replications in the simulation (Pek and Flora, 2018). Almost all interactions displayed ${\eta_{p}}^{2}$ values lower or equal to 0.02, except the two-way interaction model × amount of wording effects, which had a larger but still small effect size (${\eta_{p}}^{2}$ = 0.05, *p* < 0.001). Panel A in Figure 6 displays this interaction, which has a similar trend to the one described in Study 1: Both models performed similarly with 20% of item verification difficulty, but they tended to display some negligible differences in favor of the 1F model. The maximum difference that both models showed regarding the mean total recovery was 0.01, which is negligible, with the greatest amount of item verification difficulty. The performance of both models gradually deteriorated as the amount of wording effect increased.

The middle and lower sections of panel A in Figure 6 show the recovery of the substantive factor scores for inconsistent and consistent respondents, respectively, across the two-way interaction of model × amount of wording effects. These results mirrored those obtained in Study 1 for the wording effect of carelessness, with the recovering being approximately perfect for the consistent respondents and increasingly poorer with greater wording effects for the inconsistent respondents.

**Figure 7:**
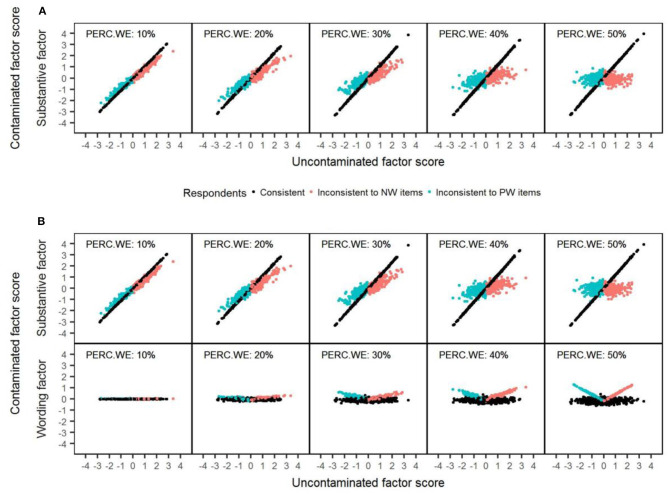
Example of recovery of the substantive factor scores with the 1F and RIIFA models in the presence of item verification difficulty. The data represented corresponds to simulated unidimensional data sets with 1,000 respondents and 20 variables. 1F, unidimensional model with one substantive factor; RIIFA, random intercept item factor analysis model with one substantive factor and one wording method factor; PERC.WE, amount of wording effect; NW, negatively worded; PW, positively worded. **(A)** 1F model. **(B)** RIIFA model.

As in Study 1, the results from a simulated sample data matrix with 1,000 respondents and 20 items were used to obtain a series of scatterplots to illustrate the relationship between the uncontaminated and contaminated substantive scores across different amounts of wording effects (see Figure 7). The trends observed for consistent respondents (colored in black) and inconsistent respondents (colored in blue or red, depending on whether they misresponded to PW or NW items, respectively) with both models mirrored the ones found in the case of carelessness (see Study 1). The correlation between the uncontaminated substantive scores and the estimated wording factor scores was also represented for the RIIFA model. For examinees who misresponded to NW items, wording scores related positively with the uncontaminated substantive score, and this relation was stronger as the amount of item verification difficulty was greater. Contrarily, for examinees who misresponded to PW items, wording scores related inversely to the uncontaminated substantive score, and the magnitude of such correlation was higher with higher amounts of wording effect.

### Structural Validity

Panel B of Figure 6 shows the regression coefficients associated with the substantive factor for both models and the wording factor of the RIIFA, as well as the proportion of explained variance by each model (*R*^2^). In terms of the mean regression coefficient for the substantive factor, both models showed a tendency to produce downwardly biased estimates, on average, with higher amounts of wording effects. Additionally, the regression coefficient associated with the wording factor had a mean of zero across conditions. Both models tended to underestimate the proportion of variance as the amount of wording effects increased, and, although the RIIFA was slightly more superior than the 1F model, the gains in variance explained were minimal.

## Study 3: Impact of Acquiescence on Parameter Estimation

In this study, Monte Carlo methods were employed to systematically assess the impact of the wording effect of acquiescence in the performance of the 1F and RIIFA models.

## Method

The study design and the procedure to generate the uncontaminated sample data matrices (see Figure 1) were similar to those described previously in studies 1 and 2. In this case, to simulate acquiescent respondents, we assumed that these individuals would select fewer response categories, implying higher levels of disagreement (1 and 2) than response options, representing higher levels of agreement (3 and 4). Thus, for the inconsistent respondents, we arbitrarily assigned to each response category a different probability of being changed, so that inconsistent respondents were generated by switching more answers with 1 than answers with 2 and more answers with 2 than answers with 3. The answers with 4 were not modified (this response option implied the highest level of agreement). The probabilities of being selected for change for response categories 1, 2, and 3 were 0.50, 0.33, and 0.17, respectively. Once a response category was selected to be changed for an inconsistent respondent, its values were modified in the following manner: 1 = 3, 2 = 3 or 4 (being the two values equally likely), and 3 = 4. Item responses were changed for each inconsistent simulee until reaching the corresponding amount of wording effect. Acquiescent respondents were selected, using the *sample* () function from the base R package (R Core Team, 2018). Finally, the same assessment criteria of the two prior studies were used to evaluate the performance of both models.

## Results

### Convergence Rates

As in Studies 1 and 2, only the RIIFA model produced solutions that did not converge, with an overall convergence rate of 94.82%. The pattern of convergence rates was similar to that found in Study 1: Non-convergence occurred with low amounts of wording effects (10 or 20%) and tests with 10 or 20 items. Convergence rates improved with larger tests and higher amounts of wording effects: 31.00% (PERC.WE = 10%, 10 items), 93.67% (PERC.WE = 10%, 20 items), and 97.67% (PERC.WE = 20%, 10 items).

### Model Fit

**Figure 8:**
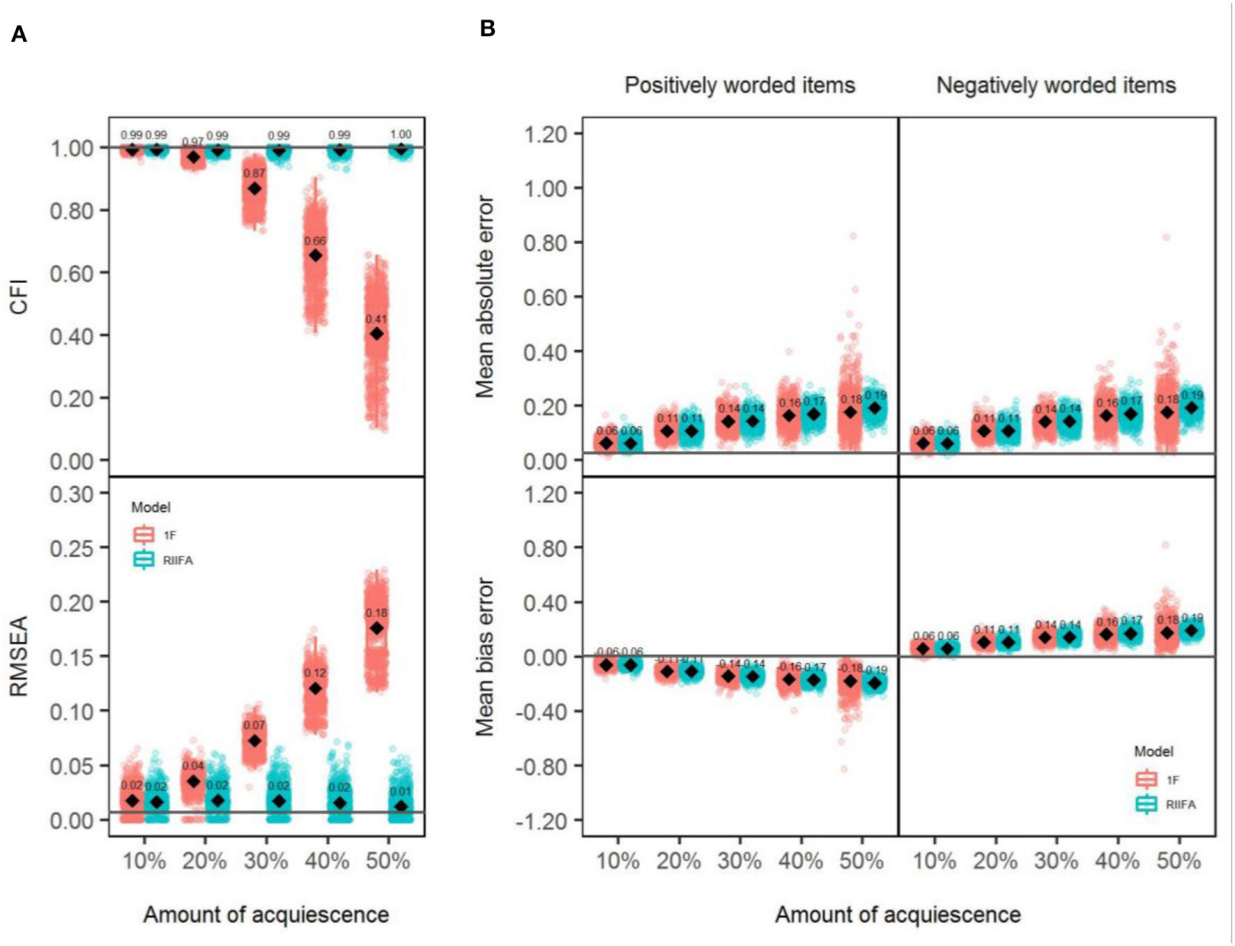
Model fit and recovery of substantive factor loadings for the 1F model and the RIIFA model in the presence of acquiescence. 1F, unidimensional model with one substantive factor; RIIFA, random intercept item factor analysis model with one substantive factor and one wording method factor. In **(A)**, the horizontal gray lines represent the mean CFI and RMSEA values for the condition with 0% of wording effect. In **(B)**, the horizontal gray lines represent the mean absolute error and the mean bias error on average for the condition with 0% of wording effect.

Panel A in Figure 8 shows the CFI and RMSEA values obtained with both models through all the simulated sample data matrices across different amounts of wording effects. Results were similar to those found in Studies 1 and 2. Both models performed similarly, showing a perfect fit, with lower amounts of acquiescence. However, as the amount of wording effect increased, the differences were more notable in favor of the RIIFA model, which consistently showed an almost perfect model fit, whereas the 1F model gradually presented a poorer fit.

### Recovery of the Substantive Factor Loadings

Panel B in Figure 8 shows the individual MBEs and MAEs for the simulated sample data matrices obtained with both models (1F, RIIFA) for each type of item (PW, NW) across the different amounts of wording effect. Looking at the $\bar{MAE}$, in general, both models produced similar estimates between them and for both types of items with any amount of wording effect, except with 40% or more of wording effect where the RIIFA model was slightly less accurate than the 1F. As in Studies 1 and 2, in general, estimates with both models were gradually less precise with higher percentages of misresponded items. As in previous studies, the mean MBE values showed that both models tended to underestimate the factor loadings of any type of item, and that this tendency increased with higher amounts of wording effects.

### Recovery of the Substantive Factor Scores

**Figure 9:**
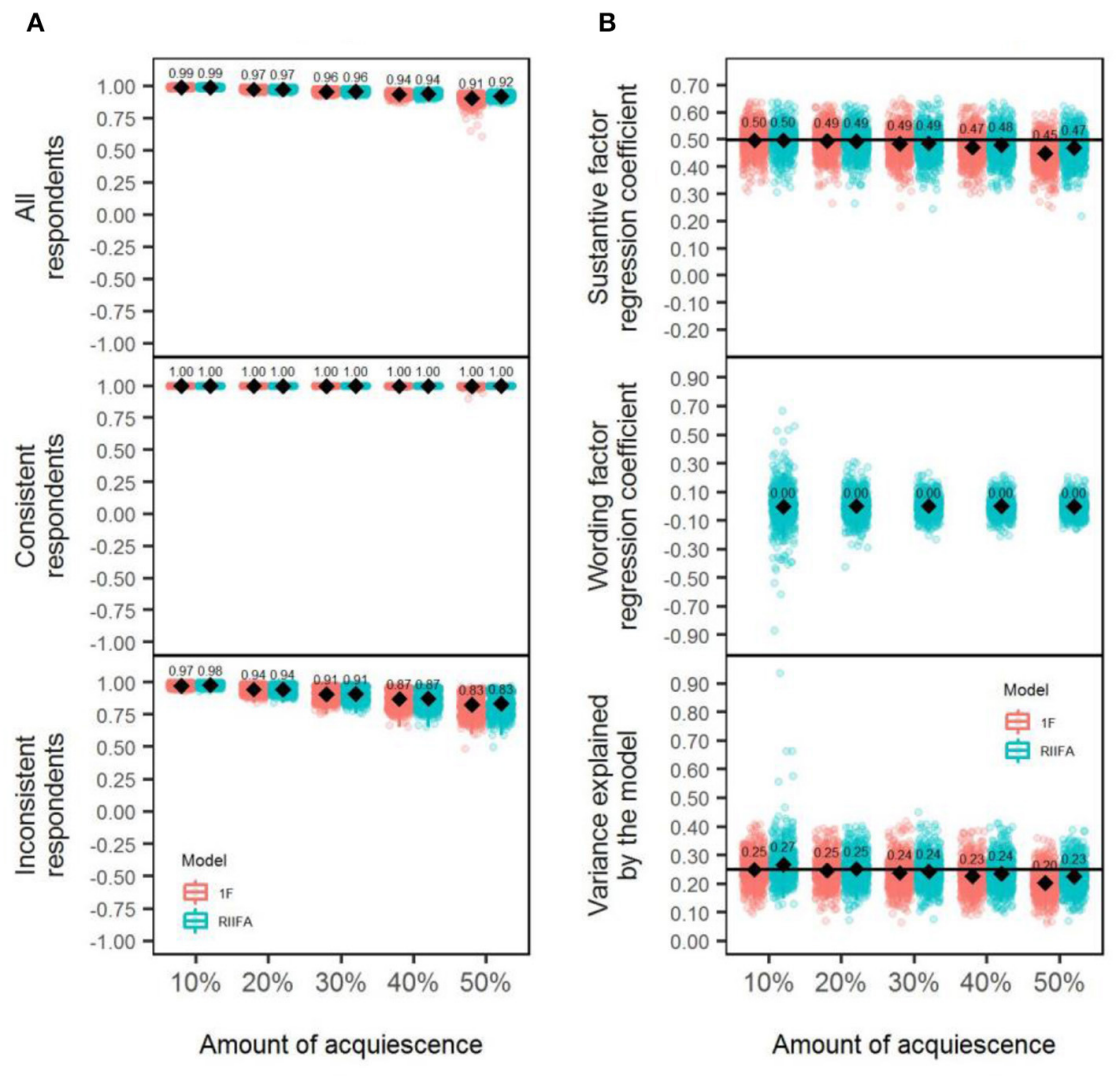
Recovery of substantive factor scores and estimation of structural validity with the 1F model and the RIIFA model in the presence of acquiescence. In **(A)**, represented values are Pearson correlations, and results of the recovery of uncontaminated scores are presented for all the respondents, separately for consistent respondents, and separately for inconsistent respondents. 1F, unidimensional model with one substantive factor; RIIFA, random intercept item factor analysis model with one substantive factor and one wording method factor. In **(B)**, the horizontal black lines represent the substantive factor regression coefficient and the variance explained by the model with 0% of wording effect.

To evaluate the differences between models, a mixed ANOVA was performed with the same specifications as in Studies 1 and 2 (Table 2, column acquiescence). Similar to Study 2, a large effects size (${\eta_{p}}^{2}$ = 0.2, *p* < 0.001) was associated with the differences in performance between models, but it had no practical relevance, because, on average, the overall recovery was similar for both models (0.95 in both cases). This large effect size emerged because of the low variability of the individual results for all the replications in the simulation (Pek and Flora, 2018). Only the two-way interaction model × amount of wording effects reached a large effect size (${\eta_{p}}^{2}$ = 0.24, *p* < 0.001), whereas the remaining interactions had ${\eta_{p}}^{2}$ values lower or equal to.04. The upper section of panel A in Figure 9 displays this interaction, which has a similar trend to the one described in prior studies. Both models performed similarly with 20% of acquiescence, and they displayed very small differences (0.01) in favor of the 1F model with 50% of acquiescence. These differences had no practical relevance, and the large effect size emerged because of the low variability of the individual results for the replications in each condition represented in panel A of Figure 9 (Pek and Flora, 2018). The performance of both models gradually deteriorated as the amount of wording effect increased but to a lesser degree than what was observed for carelessness or item verification difficulty.

The middle and lower sections of panel A in Figure 9 show the recovery of the substantive factor scores for inconsistent and consistent respondents, respectively, across the two-way interaction model × amount of wording effect. Results for both consistent and inconsistent respondents are similar to those described in Studies 1 and 2 for the wording effects of carelessness and item verification difficulty, respectively.

**Figure 10:**
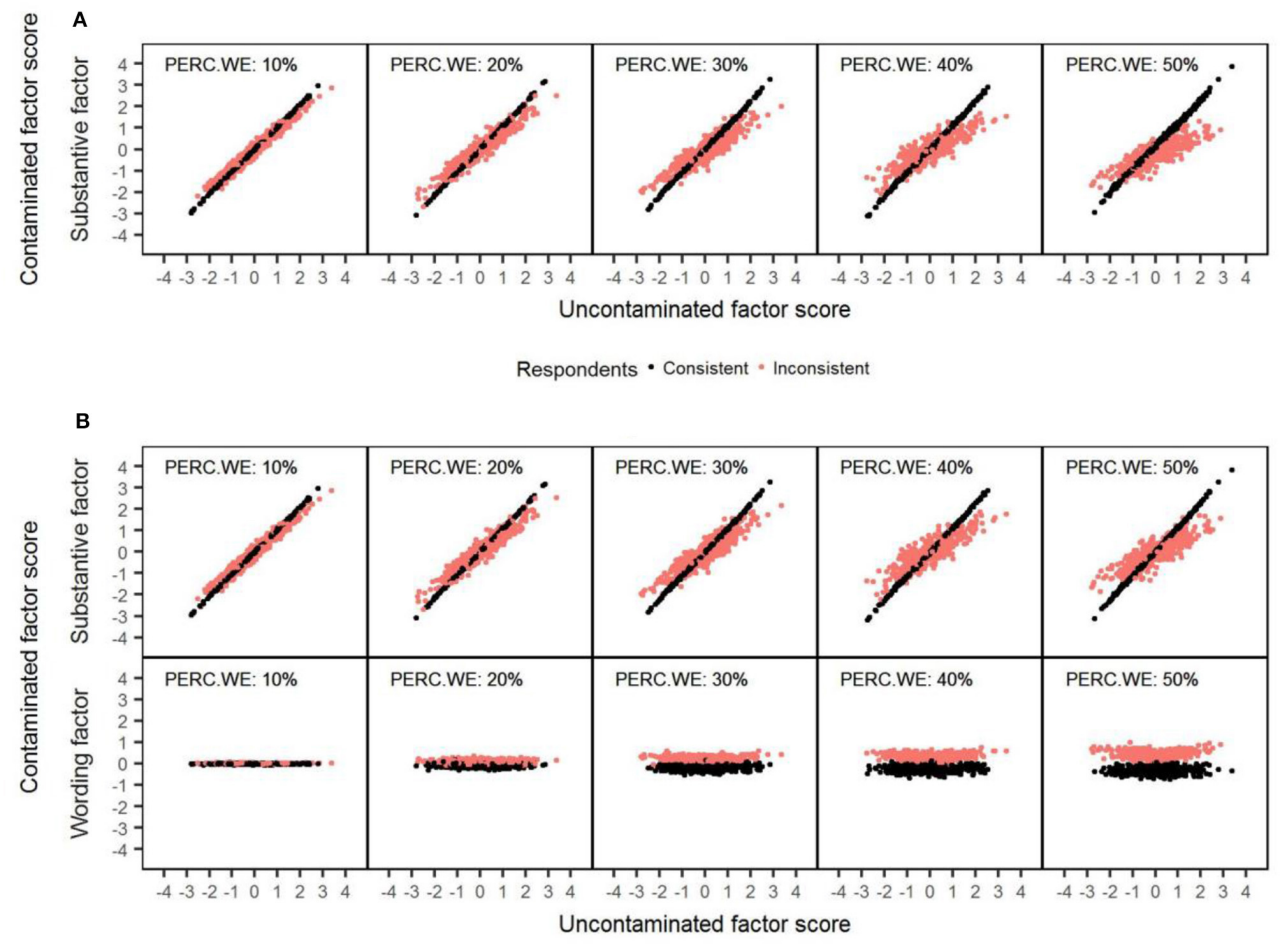
Example of recovery of the substantive factor scores with the 1F and RIIFA models in the presence of acquiescence. The data represented corresponds to simulated unidimensional data sets with 1,000 respondents and 20 variables. 1F, unidimensional model with one substantive factor; RIIFA, random intercept item factor analysis model with one substantive factor and one wording method factor; PERC.WE, amount of wording effect. **(A)** 1F model. **(B)** RIIFA model.

As in Studies 1 and 2, a simulated sample data matrix with 1,000 respondents and 20 items was used to obtain a series of scatterplots to illustrate the relationship between the uncontaminated and contaminated substantive scores across different amounts of wording effects (see Figure 10). The trends observed for consistent respondents (colored in black) and inconsistent respondents (colored in red) with both models mirrored the ones found in the case of carelessness and item verification difficulty (see Studies 1 and 2). However, in this case, the shift produced in the contaminated score estimates for inconsistent respondents was less pronounced with both models, and, therefore, the estimates were notably more accurate. This is because, in this case, items responses are modified proportionally for these respondents, while, in the cases of carelessness and item verification difficulty, item responses are not modified proportionally, since they are changed by their corresponding inverse response option (1 = 4, 2 = 3, etc.). Regarding the correlation between the wording method factor scores and the uncontaminated factor scores, the results for the consistent respondents were similar to those found in Studies 1 and 2 (there was no correlation), but the mean method factor scores were different from zero in this case. In contrast, in the case of the inconsistent respondents, the results were different from those from Studies 1 and 2, as the wording method factor scores were not correlated with the uncontaminated factor scores.

### Structural Validity

Panel B in Figure 9 shows the regression coefficients associated with the substantive factor for both models and the wording factor of the RIIFA, as well as the proportion of explained variance by each model (*R*^2^). In terms of the mean regression coefficient for the substantive factor, both models showed a similar trend to the ones displayed in Study 2, with the difference that, in this case, the two were highly accurate even with high amounts of acquiescence. Additionally, the regression coefficient associated with the wording factor had a mean of zero across conditions, similarly to Study 2. Further, both models tended to underestimate the proportion of variance as the amount of acquiescence increased, but the underestimation was noticeably smaller than for item verification difficulty (both models) or carelessness (1F model).

## Study 4: Empirical Example

We sought to compare the performance of the 1F and RIIFA models with empirical data, containing balanced scales, contaminated with wording effects that predicted a relevant outcome that was not contaminated, as specified in our simulation studies. For this task, we selected a database containing balanced scales that were intended to predict undergraduate grade point average (GPA). The constructs measured by these predictors were effort regulation, conscientiousness, and self-esteem, which have found to be positively related with undergraduate GPA (Credé and Phillips, 2011; Komarraju et al., 2011; Aspelmeier et al., 2012). In particular, the meta-analysis of Credé and Phillips (2011) found a 0.40 correlation between effort regulation and GPA for a total of 19,900 students evaluated. The GPA variable was chosen as the outcome variable because it mirrored the characteristics of our simulation, as it was a single objective variable that was not composed of PW or NW items and thus could not be affected by wording effects.

## Method

### Participants

The sample was composed of 329 undergraduate students from the University of Tarapacá, Chile, of which 160 were male (48.6%) and 169 were female (51.4%). The ages of the participants ranged from 18 to 48 years (M = 19.88, SD = 3.678). In terms of the degrees that the students were pursuing, the majority were studying engineering (30.4%), education (18.8%), medical technology (14.9%), and psychology (9.4%). The rest of the participants were studying nutrition (4.6%), language (4.0%), medicine (3.6%), multimedia design (3.6%), nursing (3.3%), ophthalmology (3.3%), social work (2.4%), and kinesiology (1.5%).

### Measures

#### Effort Regulation

Effort regulation scale was measured, using a Spanish adaptation of the Motivated Strategies for Learning Questionnaire (MSLQ; Pintrich et al., 1991). This version was created based on the guidelines of the International Testing Commission (ITC Guidelines for Translating and Adapting Tests, 2005; Muñiz et al., 2013). Three native Spanish speakers with an advanced level of English (i.e., proficiency level C1 according to the Common European Framework), and academic training in methodology and personality (i.e., doctoral studies in measurement and assessment) converged on a single version that considered linguistic and cultural factors.

The effort regulation scale of the MSLQ is a balanced scale that contains two PW items (e.g., “I often feel so lazy or bored when I study for this class that I quit before I finish what I planned to do.”) and two NW items (e.g., “I work hard to do well in this class even if I don't like what we are doing”). The items were responded, using a five-point scale, from *nothing agree* to *totally agree*. Cronbach's alpha reliability for the self-esteem scores of this study was 0.664.

#### Conscientiousness

Conscientiousness was measured, using a Spanish version of the International Personality Item Pool (IPIP; Goldberg et al., 2006). The conscientiousness scale of the IPIP is balanced, containing five PW items (e.g., “Pay attention to details.”) and five NW items (e.g., “Shirk my duties.”). The items were responded, using a five-point Likert scale that went from *nothing agree* to *totally agree*. Cronbach's alpha reliability for the conscientiousness scores of this study was 0.807.

#### Self-Esteem

Self-esteem was measured, using a Spanish version of the Rosenberg's Self-Esteem Scale [RSES (Rosenberg, 1965; Martín-Albo et al., 2007)]. The RSES is a balanced scale composed of five PW items (e.g., “I feel that I have a number of good qualities.”) and five NW self-appraisals (e.g., “At times, I think I am no good at all.”). The items were responded through a five-point Likert scale that went from *nothing agree* to *totally agree*. Cronbach's alpha reliability for the self-esteem scores of this study was 0.857.

#### Grade Point Average

The scale of academic performance in Chile ranges from 1 to 7; in this sample, the GPA ranges from 3.27 to 6.73 (M = 5.28, SD = 0.639), obtained directly from their university records.

#### Procedure

The data for this study were collected from August 28, 2017 to October 11, 2017 at the University of Tarapacá. The survey with the emotion regulation, conscientiousness, and self-esteem scales was administered in group sessions that were held in the computer rooms of the university. The GPA of the students was facilitated after the administration of the survey through the digital records of the university.

#### Ethical Considerations

The Center for Innovation and Development of Teaching (CIDD, University of Tarapacá) carried out an internal study to improve human and professional capital in its region (Curricular Harmonization Performance Agreement UTA 1501, Government of Chile). The university established the appropriate ethical procedures about the informed consent of the students, collection, storage, and custody of the data.

## Results

Table 3 shows the parameter estimates for the 1F and RIIFA structural equation models (SEM) that were fitted on the empirical data to predict undergraduate GPA. In the case of the effort regulation scale, the results showed a substantial improvement in a fit for the RIIFA model over the 1F model (e.g., RMSEA = 0.021 vs. 0.093). In terms of the substantive factor loadings, both models produced comparable mean factor loadings for the PW items (0.657 vs. 0.654), but the 1F model had somewhat lower mean loadings for the NW items (0.594 vs. 0.629). Regarding the wording factor loadings for the RIIFA model, they were 0.258. Importantly, the two models produced significant structural coefficients for effort regulation of GPA that were comparable in magnitude (0.383 vs. 0.374, *p* < 0.01). In the case of the RIIFA, the wording factor did not significantly predict GPA, and the variance explained for both models was also very similar (14.7 vs. 14.0%).

**Table 3:** Parameter estimates for the predictive SEM models of grade point average.

**Variable**	**Model fit**					**Standardized model parameters**					
**Model**	**χ^**2**^**	***df***	**CFI**	**TLI**	**RMSEA**	**FLsfp**	**FLsfn**	**FLwf**	**RCsf**	**RCwf**	**R^**2**^gpa**
**Effort regulation**											
Unidimensional	19.3^**^	5	0.962	0.925	0.093	0.657	0.594	*n/a*	0.383^**^	*n/a*	0.147^**^
RIIFA	3.4	3	0.999	0.996	0.021	0.654	0.629	0.258	0.374^**^	0.016	0.140^**^
**Conscientiousness**											
Unidimensional	410.5^**^	44	0.887	0.859	0.159	0.701	0.506	*n/a*	0.165^**^	*n/a*	0.027
RIIFA	132.7^**^	42	0.972	0.964	0.081	0.679	0.522	0.317	0.173^**^	−0.145*	0.051*
**Self-esteem**											
Unidimensional	362.0^**^	44	0.944	0.930	0.148	0.618	0.778	*n/a*	0.054	*n/a*	0.003
RIIFA	150.6^**^	42	0.981	0.975	0.089	0.632	0.756	0.280	0.049	−0.115	0.016

*N = 329 for all models. χ^2^, chi-square; df, degrees of freedom; CFI, comparative fit index; TLI, Tucker-Lewis index; RMSEA, root mean square error of approximation; FLsfp, average factor loading on the substantive factor for the positive items; FLn, average factor loading on the substantive factor for the negative items; FLwf, factor loading on the wording factor; RCsf, regression coefficient for the substantive factor; RCwf, regression coefficient for the wording factor; R^2^gpa, grade point average variance explained; n/a, not applicable; RIIFA, random intercept item factor analysis*.

^**^p < 0.01, ^**^p < 0.05.

Regarding the predictor variable conscientiousness, the fit of the RIIFA model was, again, considerably better than that of the 1F model (e.g., CFI = 0.972 vs. 0.887), with important loadings on the wording factor (0.317). In terms of the mean substantive factor loadings, the 1F model had moderately higher loadings for the PW items (0.701 vs. 0.679), and moderately lower loadings for the NW items (0.506 vs. 0.522). Again, the structural coefficients of conscientiousness on GPA were comparable for both models (0.165 vs. 0.173, *p* < 0.01), with the RIIFA model also producing a significant coefficient for the wording factor (−0.145, *p* < 0.05), which led to a somewhat higher amount of variance explained (5.1 vs. 2.7%).

Lastly, in the case of self-esteem, the RIIFA model also showed a notable improvement in the fit over the 1F model (e.g., TLI = 0.981 vs. 0.944), with associated wording factor loadings of 0.280. For this case, the 1F produced lower mean substantive loadings for the PW items (0.618 vs. 0.632) and higher mean substantive loadings for the NW items (0.778 vs. 0.756). Notably, despite the wording variance accounted by the RIIFA model, the structural coefficients of the two models were approximately equal (0.054 vs. 0.049, *p* > 0.05). In all, the results of the SEM models for the three predictors of GPA showed that accounting for wording effects improved the fit of the models considerably but did not lead to noticeable greater structural coefficients for the substantive factors. These results are in line with those of Studies 1 to 3, which showed that, while the RIIFA was greatly superior to the 1F in terms of the model fit, when wording effects were high, it did not produce less-biased levels of structural validity or a better recovery of the person factor scores.

## Discussion

The presence of wording effects is still ubiquitous in psychological measurement. This is evidenced in the fact that researchers continue proposing and testing different strategies for controlling method effects due to inconsistent responding to polar opposite items (e.g., Kam, 2018; Kam and Fan, 2018; Plieninger and Heck, 2018). Recent research has highlighted the scarce existence of systematic studies, evaluating the impact of response biases in psychometric analysis and the need to perform Monte Carlo simulation studies to shed light to this matter (Plieninger, 2016). In particular, more studies are required to evaluate whether uncontaminated true person scores can be adequately estimated in the presence of wording effects. Moreover, despite the popularity of the RIIFA model (Billiet and McClendon, 2000; Maydeu-Olivares and Coffman, 2006), little is known about its behavior to estimate person scores that may be affected by wording effects. Therefore, the current study sought to fill these gaps by systematically evaluating the performance of the RIIFA model in estimating the uncontaminated person scores (and other parameters) under the influence of three wording effects: carelessness, item verification difficulty, and acquiescence (Swain et al., 2008; Weijters et al., 2013).

### Main Findings

An initial consideration when applying the RIIFA approach concerns model convergence. Results suggested that the model has difficulty to disentangle wording and substantive variance if there is little information in the data set (e.g., few items) and the amount of wording effects is small. Usually, a model is of no use if the estimation does not converge (Forero et al., 2009). However, in this case, it may be indicating that the impact of wording effects is minimal, and thus it is not necessary to include the random intercept in the estimated model. This is valuable information.

A fundamental step in model testing is the evaluation of model fit. In terms of the RMSEA and CFI values, the RIIFA model was consistently the best approach with any type of wording effect for two reasons: it was systematically superior to the 1F model across all the conditions and always showed a good fit according to the conventional cutoff values. This is consistent with prior literature, showing that the RIIFA model is superior in terms of a model fit to models that only include substantive factors but not a wording factor (e.g., Billiet and McClendon, 2000; Maydeu-Olivares and Coffman, 2006; Abad et al., 2018; Yang et al., 2018). In fact, the fit of the RIIFA was close to the fit obtained for the 1F model with the uncontaminated datasets, indicating that it was able to properly account for the variance in the data. In contrast, but consistent with prior research (Woods, 2006), the fit of the 1F model deteriorated considerably in the presence of any type of wording effect as the amount of inconsistent responses increased in the dataset.

Regarding the recovery of the substantive factor loadings, in general, both models showed a tendency to underestimate the factor loadings in absolute value of both PW and NW items, with any type of wording effect. In terms of the accuracy, differential trends were observed according to the type of wording effect. First, for carelessness (Study 1), both the 1F and RIIFA models showed a tendency to produce estimates biased to a greater extent for the NW items than for PW items. This was expected because we simulated carelessness, specifically to NW items, as in previous research (Schmitt and Stults, 1985; Woods, 2006). That trend was accentuated with higher percentages of misresponded items. Moreover, the 1F model generally produced slightly more accurate estimates for PW items than the RIIFA, while the RIIFA model was more precise with NW items than the 1F model. All the mentioned above is valid except when examinees respond in a careless way to all the items of one type (in this case, NW items), as both the 1F and RIIFA models will be unable to distinguish which group of items is problematic, and, therefore, they will produce equally biased estimates for the PW and NW items.

Second, in the presence of item verification difficulty (Study 2) and acquiescence (Study 3), both models generally produced equally accurate estimates between them and for both types of items. This can be explained because both wording effects were simulated in a balanced way: In the case of item verification difficulty, an exact half of the inconsistent respondents misresponded to PW items and the other half to NW items. To simulate acquiescence, inconsistent respondents were randomly selected so that item responses were changed for subjects of all trait levels. As responses to PW items are mostly changed for simulees with lower trait levels, and responses to NW items are mostly changed for simulees with higher trait levels, this produces a similar bias across both types of items. Overall, both models were more accurate with lower amounts of wording effects.

The current study focuses on the recovery of the substantive factor scores. The results revealed that, with any type and amount of wording effect, both the 1F and RIIFA models systematically produced accurate person score estimates for consistent respondents. This did not occur in the case of inconsistent respondents, for whom both models produced increasingly biased estimates as the amount of wording effect was greater. This differential performance across consistent and inconsistent respondents is explained because, in the three studies here presented, we always simulated data matrices where the majority of the responses to the PW and NW were consistent with the 1F population model. This is what the estimated substantive factor reflects with both models. In the case of the RIIFA model, this was surprising because we expected that controlling for wording effects would lead to better person score estimates. In addition to the aforementioned results, the recovery of the substantive scores of inconsistent respondents was notably better with both models when the wording effect was acquiescence. This was particularly noticeable in conditions with stronger wording effects.

Furthermore, the 1F and RIIFA models performed similarly in recovering the substantive scores of inconsistent respondents when the wording effect was acquiescence or item verification difficulty. However, in the case of carelessness, the 1F model was slightly superior to the RIIFA. This differential performance is related to the recovery of the factor structure because each individual item will contribute to the estimated person score proportionally to the magnitude of its substantive factor loading. In other words, when scoring a careless person who has misresponded to NW items, the 1F model will give slightly more (but not exclusive) importance to PW items (which contain correct information about the true trait levels of inconsistent examinees) than the RIIFA. In turn, the RIIFA model will give more (but not exclusive) importance to NW items (which contain wrong information about the true trait level of inconsistent respondents) than the 1F model.

A notable finding from the three studies performed is that using the RIIFA to model wording effects produced similar results in terms of the recovery of the structural validity as “doing nothing.” These results are consistent with prior research: Yang et al. (2018) applied a depression scale to a sample of Chinese adolescents, and they found out that the model fit improved substantially when applying the RIIFA. However, they found out that the diagnostic accuracy of the instrument was slightly better when using the raw sum scores (which would be similar to “doing nothing”) than with the factor scores obtained, using the RIIFA model. Maydeu-Olivares and Coffman (2006) found similar results.

A notable finding from Studies 1 and 2 is that, in the presence of carelessness or item verification difficulty, the wording factor scores of the inconsistent respondents may reflect their uncontaminated substantive scores. This may have important implications in practice because it is common that researchers examine and interpret the correlation between a wording method factor and other measures to test validity, to identify the underlying wording effect, or even to comprehend the substantive meaning of the wording factor (e.g., Billiet and McClendon, 2000; DiStefano and Motl, 2006; Ye, 2009; Tomás et al., 2013; Alessandri et al., 2015). Therefore, we strongly recommend researchers to be especially cautious about such practice because one usually does not know the content of the wording scores.

### Limitations and Future Research Lines

The study has some limitations that deserve further discussion. First, for each type of wording effect, we developed a particular simulation strategy. Although some of the approaches were congruent with those of high impact studies from the factor-analytic literature of wording effects (e.g., Schmitt and Stults, 1985; Woods, 2006), other simulation strategies could be employed. Second, although in each simulated data set only one type of wording effect was generated separately to control for other influences, in practice, some of them can manifest simultaneously. That is, in an empirical sample, there may exist differences at the between-respondent level regarding the type of wording effect influencing the response process (Grønhaug and Heide, 1992). In addition, the responses of the same examinee may be affected by different types of wording effects simultaneously (i.e., there may be different wording effects at the within-respondent level). Another limitation of this study is that we simulated balanced scales containing the same number of PW and NW items, as has been widely recommended (e.g., Paulhus, 1991). However, prior research has shown that including different number of positive and negative items may affect parameter estimates (e.g., Plieninger, 2016). Future studies should investigate whether the RIIFA model is also robust with unbalanced scales containing fewer PW or NW items. Finally, we simulated PW and NW items with equally large (uncontaminated) population loadings. Although the literature suggests that the RIIFA method is robust to differential loading magnitudes for the PW and NW items (Savalei and Falk, 2014), we recommend that future studies determine what impact (if any) these differential loadings might have on the dependent variables considered here.

### Practical Implications

The findings from the simulation and empirical studies show that using the RIIFA to model wording effects on unidimensional balanced scales does not produce uncontaminated person scores or unbiased structural validity. Indeed, for both criteria, the “do-nothing” approach led to practically identical results. Therefore, researchers who employ the RIIFA model should be aware that doing so does not lead to unbiased person scores or structural validity. Nevertheless, we recommend that practitioners continue using this model to account for the wording effects variance in their data for several reasons. On one hand, the do-nothing approach leads to bad a model fit when the amount of wording effects is relevant. Due to this, researchers are likely to alter their substantive model (e.g., adding a new substantive factor) in others to adequately account for their data. This would lead to less interpretable models that could negatively impact theoretical developments. On the other hand, it is possible that, with multidimensional structures, the RIIFA model produces less biased estimations of the factor scores and structural validity. This is because with the “do-nothing” approach, variables may group incorrectly (e.g., all the negative items from the different theoretical dimensions may load together). Indeed, previous research has shown that the RIIFA model performs well in recovering multidimensional loading structures in the presence of wording effects, while the “do-nothing” approach leads to less interpretable loading patterns (Aichholzer, 2014; de la Fuente and Abad, 2020).

Another issue that may happen in practice is that the RIIFA model leads to a non-converged solution. According to the results of the simulation studies, a non-converged RIIFA model likely indicates that the wording effects variance is very small and can be ignored. Therefore, in these cases, where the RIIFA model does not converge, we recommend that a substantive-only factor model be estimated instead.

The findings from this study demonstrated that the RIIFA can successfully model the method variance generated from different types of wording effects that are not necessarily acquiescence. This is important because researchers often erroneously interpret that wording factors measure acquiescence effects exclusively (Billiet and McClendon, 2000). The random intercept allows modeling the individual use of the response scale (Maydeu-Olivares and Coffman, 2006), which might be influenced by different wording effects, including acquiescence. In this regard, we recommend that researchers be cautious when interpreting the relationships (or lack thereof) between wording factors and other measures because one might not be sure about the actual meaning or origin of these scores.

In closing, it is important to emphasize that fitting the random intercept model is not the only solution to explore wording effects. Alternative models should be tested and parameter estimates should be examined. But, more importantly, we strongly echo the recommendations of other researchers that the conclusion regarding the adequacy of factor models should be based not only on statistical criteria but also on substantive and theoretical considerations (Maydeu-Olivares and Coffman, 2006; Garrido et al., 2016).

## Data Availability Statement

The datasets presented in this article are not readily available because this data are property of the Center for Innovation and Development of Teaching (CIDD, University of Tarapacá) which carried out an internal study to improve human and professional capital in its region (Curricular Harmonization Performance Agreement UTA 1501, Government of Chile). Requests to access the datasets should be directed to Agustín Martínez-Molina, agustin.martinez@uam.es.

## Ethics Statement

The studies involving human participants were reviewed and approved by Center for Innovation and Development of Teaching (CIDD, University of Tarapacá). The patients/participants provided their written informed consent to participate in this study.

## Author Contributions

The original idea and the design for the simulation study were proposed by FA, LG, and MN. The data simulation codes, the statistical analyses of the simulation study, and the writing of the first draft of the manuscript were carried out by MN under the supervision of FA. LG and AM-M designed and carried out the empirical administration, data screening, performed the statistical analyses related to the empirical example, and contributed accordingly in the manuscript preparation. All the authors reviewed and approved the final version sent for publication.

## Conflict of Interest

The authors declare that the research was conducted in the absence of any commercial or financial relationships that could be construed as a potential conflict of interest.
